# New Insights and Strategies in the Nutritional Reformulation of Meat Products Toward Healthier Foods

**DOI:** 10.3390/molecules30122565

**Published:** 2025-06-12

**Authors:** Pablo Ayuso, Pascual García-Pérez, Gema Nieto

**Affiliations:** Department of Food Technology, Nutrition and Food Science, Veterinary Faculty, Regional Campus International Excellence “Campus Mare Nostrum”, University of Murcia, 30100 Murcia, Spain; pablo.ayuson@um.es (P.A.); pascual.garcia@um.es (P.G.-P.)

**Keywords:** meat, prebiotic, natural extract, food additives, oxidation

## Abstract

Meat plays a key role in human nutrition, providing protein of high digestibility and essential micronutrients. However, according to the FAO and WHO, excessive consumption of red and processed meats may increase health risks due to their content of saturated fats, sodium, and E-number additives. For this reason, recent research has focused on the nutritional reformulation of meat products to develop functional and health-promoting alternatives that meet consumer expectations and respond to market trends for healthier and more sustainable foods. However, the addition or elimination of traditional ingredients in meat products leads to problems such as changes in texture, color, or sensory acceptability that must be solved. This review will focus on current reformulation strategies in the meat industry, including the reduction or replacement of animal fat with vegetable oils using technologies such as microencapsulation, or the elaboration of 3D gels using organogelants and hydrocolloids; the replacement of the umami flavor of salt with extracts from seafoods and mushrooms; the replacement of E-number additives with antioxidant and preservative extracts from plants and herbs; and the incorporation of dietary fiber through fruit peels and vegetable by-products.

## 1. Introduction

Meat plays a key role in human nutrition, as it provides essential micronutrients and proteins of high biological value, containing highly digestible essential amino acids in proportions easily utilized by the human body. In addition, meat products account for the largest contribution to global vitamin B_12_ intake and have an important impact in supplying long-chain omega-3 fatty acids and various minerals in more bioavailable forms than plant alternatives (zinc and iron) [1], thereby playing a key role in human nutrition.

However, several international organizations and scientific authorities have underlined that excessive consumption of meat, especially processed and red meat, may be associated with certain health risks. However, a total exclusion of meat from the diet without proper nutritional planning can also have negative consequences for health. This is due to the high content of saturated fatty acids (SFAs) in meat (60% of total fatty acids), which can raise the plasma concentration of low-density lipoprotein cholesterol (LDL-C), thus increasing the risk factors associated with the development of cardiovascular disease (CVD) [2]. However, this relationship is subject to debate, as scientific evidence suggests that certain animal fats, such as those found in pork and beef, may also provide essential fatty acids, including arachidonic acid, which are not present in vegetable oils and play a key role in a balanced human diet [3]. In addition, unlike other sources of vegetable protein, meat has a low content of bioactive compounds with antioxidant capacity, as well as a reduced content of dietary fiber (DF), which is associated with beneficial health effects [4]. For these reasons, consumers now consider meat products to be less healthy and less appetizing, which makes them more selective about the products they consume, since they are increasingly aware of the need to improve their health through the food intake. There is a growing demand in the market for healthier and more functional foods, being one of the main driving forces behind new trends and innovations in the development of new healthy products, among which are those of meat origin [5].

Recent research has shown different types of strategies for making meat products healthier, such as genetic strategies [6] or cultured meat [7]. However, there is a wide range of alternatives that can be used in the development of meat products to reduce the negative effects of excessive meat consumption. These strategies are mainly based on the reduction or elimination of unhealthy components through substitution with a healthier alternative, which is presented as a cheaper alternative capable of maintaining the benefits of the protein, minerals, and vitamins of meat; all of this while maintaining the same technological and sensory quality criteria required for the development of healthy meat products. Recent research suggests that consumers value very positively the use of this type of strategies, both the “addition” and “reduction” of different components, even being willing to pay a higher price for these products [8]. This review article will mainly examine the possibility of using different reformulation techniques for fats, salt, dietary fiber, and E-number additives in meat products and their potential for improving human health and consumer satisfaction.

## 2. Fat Enrichment and Replacement

The consumption of meat, especially red meat, is considered intrinsically harmful socially and by various organizations due to its high SFA content. In fact, international organizations recommended that SFA intake should be less than 10% of the total caloric value [9]. However, there is some controversy about the real harm that can be caused by habitual meat consumption. Two European, four Asian, and one North American study showed no increased risk in any intake or only demonstrated an increased risk in intakes greater than 75 g/day [3]. However, efforts to modify the fat composition aim not only at reducing harmful components but also at enriching the products with healthier lipids to improve their nutritional profile. In addition, they also have a high ratio of dietary ω-6/ω-3 polyunsaturated fatty acids (PUFAs), which is nutritionally unfavorable for human health. Thus, frequent consumption of meat products can lead to inflammation and the onset of several chronic diseases [10].

Consequently, the reformulation of the fat content in meat products has emerged as a key strategy in the development of healthier meat-based foods. The main approach to improve nutritional quality and reduce the consumption of saturated fat in meat products has been achieved by enriching animal fat with vegetable and/or marine oils [11], as they can be a great option for the partial or complete replacement of SFAs and trans fatty acids [12]. It is well known that a reduction in SFA intake can lower LDL-c levels in the blood, leading to a decreased risk of cardiovascular disease. A meta-analysis revealed that the dietary intake of palmitic acid, stearic acid, and saturated fatty acids from meat and unprocessed meat has been modestly linked to an increased health risk. In contrast, the consumption of alpha-linolenic acid, long-chain omega-3 fatty acids, and linoleic acid has been moderately associated with a reduced risk [13]. Meyer et al. [14] also demonstrated that a diet rich in PUFAs, vegetable protein, and fiber may reduce visceral fat and LDL-c, independently of weight loss. Hooper et al. [15] suggested that a reduction in SFA intake provided a 21% reduction in cardiovascular events and that replacing 10% of SFAs with PUFAs decreased cardiovascular events by 27%. On the other hand, vegetable oils such as wheat germ [11], rapeseed [16], or olive oil [17] present high amounts of PUFAs compared to meat fat.

However, meat fat plays a functional role in the texture, mouthfeel, juiciness, and sensory acceptability of products [18]. Therefore, although nutritional improvements can be achieved, the substitution of animal fat with vegetable oils remains challenging due to the potential adverse effects on the organoleptic properties of the final product [19]. Potential fat substitutes must perform well in terms of nutritional value, in addition to providing good structural and sensory attributes. The techniques most commonly used for lipid reformulation of meat products are direct addition, interesterification, microencapsulation, and the use of gel and oleogel pre-emulsions. The different strategies for fat substitution in meat products are summarized in Table 1.

### 2.1. Direct Replacement

The direct replacement of meat products using vegetable oils has been used in recent years. Öztürk & Turhan [32] used pumpkin seed oil as a fat replacer and functional ingredient in meatballs. Further, different authors have tested this form of replacement in ham [33], chicken nuggets [34], and burgers [35,36].

However, although the direct enrichment of oil in meat products had a positive effect on the nutritional quality of the final product, it showed significant drawbacks. Due to the poor protection of the oils in the product, oxidation reactions were generated more frequently, shortening the shelf life of the products. Furthermore, the reduced solubility and increased hardness problems resulting from the formation of small fat globules significantly affect some quality attributes (taste, color, and sensory acceptability) of the reformulated products. For this reason, the direct substitution of animal fat is an ineffective solution if the sensory and physicochemical quality of the meat product needs to be maintained. This is the reason why, in recent years, other alternatives have been investigated that are capable of better preserving the oil and simulating the texture of animal fat.

### 2.2. Microencapsulation

One technique capable of avoiding the degradation of vegetable oils in meat products and reducing their negative sensory effects is microencapsulation. Microencapsulation is a technique in which small particles are enclosed by a coating wall and embedded in a matrix to form small capsules with desirable properties; normally, the capsules are formed using a microencapsulating agent, such as alginate, gum arabic, maltodextrin, or modified starch. Since vegetable oils are more susceptible to oxidation due to their chemically unstable nature, when such oils are exposed to moisture, oxygen, and light, they undergo oxidation, leading to the formation of volatile compounds and flavor and sensory degradation of enriched meat products. For this reason, microencapsulation can be used as a physical barrier to protect bioactive compounds present in oils from degradation reactions. In addition, encapsulation also resists high temperature conditions, as well as changes in pH and humidity, and can therefore be of interest in the reformulation of cooked and cured meat products.

Fish oil is one of the most commonly encapsulated oils that have been applied in meat products, as well as some vegetable oils such as linseed oil. The reason for the use of these oils is their high content of ω-3 fatty acids not present in meat, which have a variety of health benefits. Encapsulation is one of the best techniques for the preservation of fish oil, limiting its oxidation. Kawecki et al. [20] tested the effect of microencapsulated fish oil as an additive in poultry sausages, resulting in improved elastic properties and dynamic viscosity. In addition, more recent studies have explored the use of microencapsulation in different meat products, such as patties [21], pates [22], or sausages [37]. Despite the many advantages of microencapsulation of oils, the high cost of encapsulating agents, as well as the equipment necessary for encapsulation, limit the commercial use of this technique as a replacement for animal fat [38].

### 2.3. Interesterification

Interesterification is a process used to obtain lipids with predetermined properties. This process can be achieved by chemical or enzymatic means and involves the randomization of fatty acids by rearrangement within and between triacylglycerol molecules to obtain desirable melting characteristics, giving the possibility to obtain a wide range of lipids with modified structure and physicochemical properties [23]. This process does not alter the structure of the fatty acids, so the bioavailability of the valuable unsaturated fatty acids present in vegetable or marine oils remains unchanged.

The use of interesterification has been used to substitute fat in a wide variety of meat products. Wirkowska-Wojdyła et al. [23] observed the effect of interesterification on the thermal and technological properties of rapeseed oil and its possible use in the incorporation of meat batters. Kılıç & Özer [24] investigated the effect of replacing beef fat with interesterified palm kernel oil on the quality parameters of dry-fermented sausages and frankfurters. The conclusions of these studies were a good acceptance of the meat products and a nutritional improvement in the lipid profile, improving the ratio between n-3 and n-6 fatty acids to a more favorable level.

### 2.4. Emulsions

An emulsion is a mixture of two completely immiscible liquids, generating a colloidal dispersion by one liquid in the form of small droplets distributed in another insoluble liquid [39]. These emulsion gels have a stable structure that gives them technological properties suitable for food reformulation. There are two main types of emulsions: water-in-oil (W/O) emulsions and oil-in-water (O/W) emulsions. Both types of emulsions confer different physicochemical characteristics and their use depends on the type of oil used and the final product to be reformulated. However, O/W type emulsions are thermodynamically unstable and tend with a tendency to undergo aqueous and oily phase separation behaviors, so a suitable emulsifier is added to improve stability. These are substances with a hydrophilic and a lipophilic part whose function is to reduce the surface tension between the aqueous and oily phases when added to the emulsion, thus promoting a thermodynamically stable and homogeneous preparation.

These emulsions have a stable structure and excellent stability, which gives them technological properties suitable for food reformulation. In addition, emulsion gels exhibit a soft solid texture and can exhibit fat-like physical behavior in emulsified meat products [40]. Therefore, they have increasingly become an alternative for designing and developing healthy and functional foods, as well as a vehicle for vegetable oils in meat products. These structured emulsions can be classified into gel emulsions and oleogels.

#### 2.4.1. Emulsion Gels

One of the most innovative technologies used to replace animal fat is the use of emulsion gels or hydrogels. In this gelled structure, emulsion droplets (O/W or W/O) are contained within a hydrogel matrix. For the development of a hydrogel, a liquid emulsion is first developed and then a hydrocolloidal stabilizer is used. These polymers are used once the emulsion is formed for the correct formation of the hydrogel. During the gelation process, double helical and cross-linked helical domains are formed to form three-dimensional structures [41]. On the other hand, the gelling process can also be obtained by heating, acidification, or by employing enzymes with hydrocolloids [42]. Numerous types of proteins such as milk, soy, or egg, as well as polysaccharides (alginate, carrageenan, inulin, etc.), have been used to create a gel emulsion. The flexible characteristics of emulsion gels make it possible to introduce functional ingredients of vegetable origin, such as oils with an unsaturated fatty acid profile, minerals, and even fiber and antioxidants [43]. Furthermore, the use of hydrogel emulsions as a replacement for animal fat may be of particular interest because both hydrophilic and lipophilic substances can be incorporated, as the oil content is generally ≤50% of the total gel [44]. On the other hand, other positive functions of emulsion gels have been discovered, since due to their 3D structure they are able to improve cooking performance, oxidative stability, and even sensory properties of reformulated products.

In recent years, the use of emulsion gels with a wide range of vegetable and marine oils has been described. It has been possible to replace animal fat by using emulsion gels with chia [45], chestnut [46], grape seed [25], linseed [47], microalgae [48], tiger nut [27], or wheat germ oils [11]. In general, these oils are used as an alternative to the inadequate lipid profile of meat; oils such as linseed and olive have a higher proportion of PUFAs and monounsaturated fatty acids, and increased ω-6/ω-3 PUFAs and PUFA/SFA ratio [17].

In general, the application of these hydrogels had a positive consumer acceptance by simulating the texture and organoleptic characteristics of animal fat. Câmara et al. [26] also observed good acceptability of bologna sausages using an emulsion with chia oil. A hedonic test as well as an evaluation by means of a CATA questionnaire determined a purchase intention as well as a taste, color and aroma similar to the control sausages. Another study investigated the effect of peanut and flaxseed oils as fat substitutes in fermented beef sausages, obtaining similar values for flavor, oiliness, and overall impression attributes [49]. This methodology has been tested in a wide variety of meat products such as patties [43,48], sausages [45,50,51], frankfurters [52], duck meat [28], or meat emulsions [25,53].

#### 2.4.2. Oleogels

**Figure 1:**
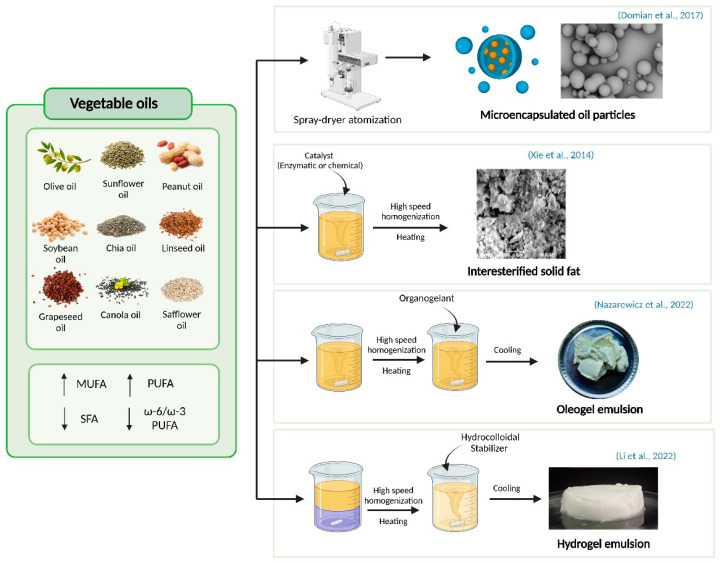
Novel strategies for the introduction of healthy oils in meat products [56,57,58,59].

Organogels are three-dimensional network structures with thermally reversible properties formed by an organic liquid and organogelants. When the organic phase of an organogel is edible oil, it is called an oleogel. Unlike emulsion gels, which are formed by gelling the continuous phase, oleogels are liquid oils that are transformed to the gel state by organogelants (Figure 1). Therefore, these emulsions present a much higher oil content than emulsified gels, reaching values of approximately 90–99% (*w*/*w*) [40]. Similar to emulsion gels, adding an organogelating agent to the emulsion changes the physical properties of the oil, converting the liquid oil into a solid substance, making the rheological behavior similar to that of fat. During the organogel formation, the interactions responsible for gelation are π-π stacking, hydrogen bonds, and electrostatic and van-der Waals interactions [54]. Due to their physicochemical properties, oleogels have recently attracted attention in the agri-food sector as a replacement for animal fat. It is a safe alternative in food products, easy to manufacture and allows the introduction of a healthier fatty acid profile in meat products. Different types of molecules have been used as organogelants, depending on the desired physical properties and their use in specific types of meat products. Sterols such as β-sitosterol and γ-oryzanol have been used as organogelants, as these molecules are able to self-assemble into tubules to form strong gels in the presence of a variety of edible oils. In addition, some proteins, such as soy protein, as well as waxes such as beeswax and rice bran wax can also play this role [19]. On the other hand, the selection of the vegetable oil used in the formulation of organogels plays a critical role in defining the final characteristics of the oleogel, determining its rheological properties and its melting and crystallization behavior. Numerous studies have been described using different vegetable oils such as linseed [29], soybean [55], or olive [31] oils.

Recently, the use of these oils in the form of oleogels has been used for the preparation of patties [30,60], meat batters [61,62], sausages [31,63], and pates [29,64]. Although the use of oleogels significantly improved the nutritional quality of the great majority of reformulated meat products, some of these studies showed a negative impact on the sensory acceptance of the final product. Gómez-Estaca et al. [65] observed a low acceptability in flavor, aroma, and color of pork patties reformulated with a curcuma oleogel. However, the major drawback of these oleogels is that the U.S. Food and Drug Administration (FDA) does not include most organogelants on the “generally recognized as safe” (GRAS) list. Therefore, a new regulation approving these oleogelants as GRAS is required for their use to be scalable in the food industry.

## 3. Sodium Reduction

Salt, or sodium chloride (NaCl), is a widely used ingredient essential to human health. Salt plays a fundamental role in fluid balance, nerve impulse transmission, and muscle contraction. Due to its preservative and flavor-enhancing properties, sodium chloride has become an indispensable ingredient in the production of many foods [66]. In meat products, it performs a variety of important functions. Its main function is to solubilize myofibrillar proteins, retaining water during processing and cooking, resulting in improved meat structure and texture. In addition, salt has antibacterial and preservative properties that reduce water activity and enhance flavor, which directly affects sensory quality and shelf life [67]. For these reasons, the reduction or substitution of salt in meat products poses techno-functional challenges that affect both the organoleptic and shelf-life properties of final products. Despite its technological functions, sodium chloride has been considered by various researchers and medical institutions to be a detrimental ingredient for health. Current evidence from cohort studies suggests a direct relationship between sodium intake and cardiovascular events and suggests that the lowest risk of death or cardiovascular disease occurs in populations consuming an average sodium intake (3 to 5 g/day) [68]. This is because a rise in plasma sodium increases osmolarity, thus inducing a shift of fluid from the intracellular to the extracellular compartment, stimulating vasopressin secretion and leading to water retention. By restoring plasma sodium to its original level, the extracellular fluid volume also increases, thus increasing blood pressure.

It has been shown that reducing salt intake (2.3 g/day) can slightly lower blood pressure in hypertensive and normotensive patients [67], decreasing the risk of CVD. However, most populations worldwide consume 3 to 6 g of sodium per day, surpassing the current recommended level of less than 2.3 g/day of sodium, which is well below the range of most of the global population. For this reason, salt reduction in foods and meat products has been a priority for more than a decade, and various strategies have been employed to address this need. Various strategies have been developed to reduce or reformulate salt in meat products using different ingredients and extracts in order to maintain the preserving properties and umami flavor provided by salt. Different NaCl reduction strategies used in the meat products reformulation are described in Table 2.

The use of chloride salts such as potassium chloride (KCl), calcium chloride (CaCl_2_), and magnesium chloride (MgCl_2_) can be a good alternative as a salt substitute. Divalent (calcium and magnesium) and monovalent (potassium) cations have been found to enhance the functionality of meat proteins during gelation. Potassium chloride has been used as a substitute for NaCl in dry-cured meat sausages, obtaining acceptable results for the growth of lactic acid bacteria and enterococci without affecting sensory attributes [71]. In addition, Teixeira et al. [69] tested the effect of KCl + sub4Salt^®^ and NaCl + KCl as a salt substitute on the physicochemical properties, chemical composition and sensory characteristics of pork sausages. This study suggested that a 2% salt substitution for the different KCl blends did not significantly affect the sensory and microbiological analysis between the different formulations. However, new alternatives to the use of KCl are being studied, as potassium ingredients are often bitter or metallic and tend to interact with other flavors to change the overall flavor of food products [83].

Seafoods, such as sea spaghetti, wakame, and nori, are rich sources of minerals, which can act as flavor enhancers, offering a promising alternative to traditional salts for flavor enhancement [76]. Seaweeds are rich in umami compounds, mainly glutamates, so they can confer depth of flavor to dishes without excess sodium. In addition, seaweed ingredients have received much interest in recent years as functional ingredients, since their addition to meat formulations can be a source of polysaccharides, thus improving the structure and resistance of reduced-fat products. On the other hand, they are also another excellent source of bioactive compounds such as proteins, fatty acids ω-3, carotenoids, phenolic compounds, vitamins, and minerals. Pindi et al. [74] studied the effect of sea moss (*Kappaphycus alvarezii*) on chicken patties. The addition of this algae at 2 and 4% increased water holding capacity (WHC) and minimized cooking loss in salt-reduced patties. The effects of including sea spaghetti and Irish wakame in pork sausages have also been observed [73]. This reformulation improved the shelf life of packaged sausages (70% N_2_, 30% CO_2_) was evaluated by lipid oxidation and total viable counts.

Vilar et al. [76] studied the inclusion of 1% of edible seaweeds (*H. elongata*, *P. umbilicalis, P. palmata*, and *U. pinnatifida*) on the chemical, sensory, and volatile components profile of pork sausages. However, seaweeds negatively affected the aroma, texture and overall acceptability parameters, with the sausages formulated with *H. elongata* being the best scored. Although seafoods provide natural salinity and can be a good substitute for NaCl in meat products, it is important to note that some varieties, especially those rich in minerals, can also have a slightly bitter taste [84] and can contribute a dark color to meat [72,73,74].

On the other hand, some fungi could be considered as a potential ingredient in the development of low-sodium food products. Different peptides (containing glutamic acid) with unique flavoring properties have been found in mushrooms and can even interact with other volatile compounds, influencing the final aroma and flavor of foods. This umami flavor arises from the harmonious interaction of several components, such as some amino acids, 5′-nucleotides, succinic acid, and umami peptides [85]. This flavoring property means that mushrooms can be used in meat products as a salt substitute. In addition, a wide range of bioactive compounds can be found in mushroom, such as fiber, polyphenols, and vitamins.

Several investigations have evaluated the effect of some fungi on meat products. Banerjee et al. [86] evaluated the impact of different amounts (2%, 4%, and 6%) of powdered enoki mushroom (*Flammulina velutipes*) stem residues on the quality and shelf life of goat meat nuggets. The inclusion of this mushroom improved the oxidative stability of treated nuggets by reducing thiobarbituric acid reactive substances (TBARS) during a 9-day refrigerated storage period. In addition, the inclusion of this extract did not adversely affect the color and sensory attributes of the treated meat nuggets. Another study tested the effect of two edible fungi (*Agaricus bisporus* and *Pleurotus ostreatus*) as a salt substitute in frankfurter-type sausages [77]. In this study, the addition of flours significantly improved the dietary fiber and protein contents, and burgers with 2.5% flour presented acceptable sensory parameters, so they could be an acceptable option to provide fat and salt reduction.

Different plants and fruits have also been studied as NaCl replacers in meat products. [80] explored the impact of NaCl (1% and 2%) and soy protein isolate (3% and 6%) on the physicochemical properties of frankfurters. The reformulated frankfurters demonstrated higher L* (lightness) and a* (redness) values than control samples, as well as acceptable texture values for the reformulated products. It has been shown that soybeans can impart an umami flavor to meat due to their amino acid composition, rich in Glu, Asp, Phe, Ala, Gly, and Tyr [87]. Finally, by-products of the wine industry have also been studied as salt substitutes in meat products; one study evaluated 2% red grape pomace as an antimicrobial agent in marinated chicken breasts [81]. Although the chicken breasts marinated with a brine of 0.5% salt and 2% seasoning had the same shelf life as those marinated with 2% salt, the reformulated chicken breasts were rated lower in sensory evaluation.

## 4. Replacement of Additives

Consumers are increasingly concerned about their personal health and well-being and are willing to pay a higher price for healthier and more natural products. In general, the concept of “natural” in a food is related to three different categories: the origin of the food, its processing (technology and ingredients), and, finally, the properties of the final product [88]. One set of ingredients that is most questioned by consumers is additives, as they are usually perceived as toxic and unrelated to the food. For this reason, in response to consumer demand, the food industry has many opportunities for innovation and the elaboration of new products within the framework of E-number additive-free products. Numerous studies have been able to eliminate preservative, antioxidant, or texturizing additives without affecting or with minimal impact on the organoleptic properties of the final product.

### 4.1. Substitution of Antioxidants

Oxidation is the most important factor in evaluating the quality and acceptability of meat products, affecting multiple attributes such as flavor, aroma, color, and texture. During meat processing and storage, various compounds can be produced such as reactive oxygen species (ROS), reactive nitrogen species (RNS), and reactive sulfur species (RSS). The main targets of these free radicals in meat products are lipids, proteins, and pigments, which are especially sensitive to the UV component of light, oxygen, storage temperature, and processing methods [89]. During the lipid oxidation process, various secondary products can be generated, in particular reactive carbonyl species such as hexanal, malondialdehyde (MDA), and 4-Hydroxynonenal (4-HNE).

One way to limit or inhibit oxidation is through the use of antioxidant additives, thus improving the quality and shelf life of products. The list of authorized antioxidants is short in the EU, but longer in the U.S. The only approved pure synthetic antioxidants on the EU list are gallates (E-310), tertiary butylhydroquinone (TBHQ) (E-319), and butylated hydroxyanisole (BHA) (E-320), which are only allowed for the processing of dried meat. However, the additives erythorbic acid (E-315) and sodium erythorbate (E-316) are also permitted for the formulation of cured meat products and canned meat products; and ascorbic acid (E-300) and sodium ascorbate (E-301) for the formulation of foie gras. However, it has been studied that the intake of some of these additives can have negative implications on human health if the established limits are exceeded. It has been shown that excessive ingestion of propyl gallate can be related to the induction of male infertility through disruption of calcium homeostasis and mitochondrial function [90]. In addition, butylated hydroxytoluene (BHT) and butylated hydroxyanisole (BHA) can cause DNA damage and mutations, increasing the formation of enzymes for metabolization in the liver, kidneys, and nerves [91]. Other studies have observed that TBHQ can lead to the formation of 8-hydroxy-deoxy-deoxydeoxyguanosine in thymus DNA due to the production of ROS such as superoxide anion [92]. For these reasons, the research for the use of antioxidants of natural origin has grown in recent years, finding numerous alternatives that are able to replace these additives.

Antioxidants of natural origin have been used in meat products from numerous spices, herbs, fruits, or vegetables; these natural extracts are able to offer a similar function to their synthetic alternatives, with the advantage that they are easy to label and more attractive to the consumer. The activity of natural extracts is mainly due to phenolic compounds, which are structurally related but differ in amount and type, depending on the specific source. The main antioxidant components of spices and herbs are phenolic acids (gallic acid, caffeic acid, and rosmarinic acid), flavonoids (catechin, quercetin, apigenin, kaempferol, naringenin, and hesperetin), phenolic diterpenes (carnosic acid and carnosol), and volatile oils (eugenol, carvacrol, thymol, and menthol).

Spices and herbs are ingredients widely used in the formulation of meat products. They are natural additives that exhibit a high antioxidant capacity and can prevent lipid oxidation of the meat, in addition to providing good acceptability to the final product. Turmeric (*Curcuma longa* L.) is one of the plants most widely used as a spice in meat, providing color and improving flavor. Chen et al. [93] tested the shelf life extension effect of turmeric in beef meatballs. This study showed that this spice delayed the degradation of the color and texture of the meatballs and protected the structure of the myofibrillar proteins, in addition to improving their sensory acceptability after storage. Another study in lamb sausages showed that turmeric was able to replace high concentrations of sodium erythorbate, inhibiting lipid oxidation after 18 days of storage [94].

Rosemary extract (*Rosmarinus officinalis* L.) is an herb with great hepatoprotective, antifungal, insecticide, and antioxidant properties. The antioxidant effect of rosemary extract has been demonstrated in many studies in different meat products such as patties [95], chicken meat [96], nuggets [97], or burgers [98]. Nieto et al. [99] studied the effect of rosemary in pork patties, showing that this extract was able to delay the loss of thiol groups, preventing protein disulfide cross-link formation. This antioxidant capacity of rosemary is mainly explained by its high content of phenolic diterpenes (carnosol and carnosic acid) that act as hydrogenic donors in the chain reaction of free radicals. Due to its great potential for meat preservation, the EU approved rosemary extract as the only natural antioxidant additive, under the name of E-492. Other herbs such as acerola (*Malpighia emarginata*) have been used in the preparation of meat products. Van Buren et al. [100] used acerola and rosemary extract in the preservation of frozen beef. The combination of both extracts delayed oxidation of the meat and improved the color after thawing, maintaining the reddish color.

On the other hand, plant and fruit by-products or non-commercialized parts rich in bioactive compounds are being investigated as natural additives in meat products for their antioxidant properties. The use of these industrial by-products is in line with the concept of circular economy and could help in reducing the environmental impact of food processing and waste production, while providing benefits to the meat industry in extending the shelf life of products. For example, Pateiro et al. [101] tested the effect of guarana seeds on pork patties, obtaining as a result that the extract is able to enhance the color of pork patties in addition to obtaining lower lipid oxidation values than control patties. Olive tree also generates a large amount of by-products for oil production. In general, these bioresidues are rich in important bioactive compounds such as 3,4-dihydroxyphenylethanol or hydroxytyrosol (HXT), showing interesting antioxidant characteristics and having beneficial effects on health. Martínez-Zamora et al. [102] tested organic hydroxytyrosol from olive leaves in lamb meat patties. This extract provided a higher antioxidant and preservative capacity in maintaining the nutritional value than the HXT of synthetic origin that was used as a control.

Finally, recent research is testing green solvents as ways of extracting bioactive compounds in natural extracts, which could be beneficial for the reformulation of meat products. The main idea is to replace traditional and potentially harmful organic solvents (hexane, benzene, methanol, chloroform, petroleum ether, and acetone) with non-toxic or food safe ones [103]. Among the most prominent techniques are accelerated solvent extraction (ASE), enzyme-assisted extraction (EAE), high hydrostatic pressure extraction (HHPE), infrared-assisted extraction (IAE), microwave-assisted extraction (MAE), pulsed electric field extraction (PEF), subcritical fluid extraction (SFE), and ultrasound-assisted extraction (UAE) [104]. These novel extraction methods not only reduce the use of harmful chemicals, but also improve extraction efficiency and selectivity, thereby preserving the bioactivity of valuable compounds. The implementation of these techniques could be of interest for the future elaboration of more sustainable meat products, while replacing harmful additives with natural extracts. The integration of these green technologies is in line with global sustainability objectives and offers a promising avenue for the development of functional meat products with improved nutritional and sensory properties. Continued technological advances in this field are expected to facilitate their widespread adoption in the food industry, which could represent an important step forward in the formulation of healthier and more environmentally friendly meat products.

### 4.2. Substitution of Food Preservatives

Sodium nitrite (NaNO_2_) is a widely used curing agent in processed meat products and is used as a preservative against microbial contamination, but also contributes to sensory attributes (formation of reddish-pink color and flavor development) [105]. Nitrite acts as an essential enzyme inhibitor at multiple sites in the bacteria simultaneously, causing a breakdown of the proton gradient that the microorganism needs to generate ATP and preventing microbial growth [106]. Through this mechanism, nitrites are able to control the growth of pathogenic bacteria such as *L. monocytogenes*, *S. aureus*, *E. coli*, *C. perfringens*, or *Salmonella* spp. [107]. For this reason, sodium nitrite is a very important and key additive for public health, preventing the development of foodborne diseases. In addition, potassium nitrite (KNO_2_) is also authorized as a preservative in meat products. It has a similar mode of action, inhibiting microbial growth, and is often used as an alternative in formulas intended to reduce sodium content. However, it is also regulated in the European Union due to its possible role in the formation of N-nitrosamines.

Nitrite is an additive regulated by the EU under the nomenclature E-249 (KNO_2_) and E-250 (NaNO_2_). However, several toxicological implications have recently been detected due to its possible involvement in the formation of highly carcinogenic nitroso compounds (N-nitrosamines) [108,109]. This has led the European Food Safety Authority (EFSA) to recently request a scientific opinion on the public health risks related to the presence of nitrosamines in food [110]. Under conditions of low pH and high temperatures, the residual nitrite present in cured meat products in combination with certain amines or amides can potentially form carcinogenic N-nitrosamines. On the other hand, throughout the gastrointestinal tract there is a possibility that nitric oxide (NO) formed by the consumption of nitrite can react with precursors of nitrogenous compounds, leading in turn to the formation of endogenous nitrosamines [111]. According to different studies, N-nitrosamines could contribute to increase the risk of several types of cancer, such as colorectal [112], renal [113], or hepatic cancer [114].

For this reason, researchers are increasingly looking for new natural alternatives that can replace the functions of nitrites without causing any possible health hazard. In recent years, numerous studies have been carried out on NaNO_2_ substitution using extracts from different plant sources such as cranberries [115], sea buckthorn [116], coriander [117], or thyme [118]. Positive results have been obtained in various meat products after nitrite substitution, Golden et al. [119] evaluated the efficacy of an antimicrobial mixture containing dry vinegar combined with fruit extracts and spices in cured ham. The spice mixture was positive against *C. perfringens*, obtaining a similar inhibition compared to traditional cured ham. Another study evaluated the substitution of sodium nitrite by spinach powder and essential oils in pork loin [120], obtaining as results a decrease in protein and lipid degradation. One of the extracts most commonly used as a substitute for sodium nitrite in meat products is tomato. This is mainly because, in addition to its antimicrobial properties, it also contributes to the development of the characteristic reddish color in cured products, thanks to its high lycopene content. Numerous researchers have reformulated several types of meat products using tomato powder or tomato residues [121,122]. These studies found an improvement in the reddening of meat products and a delay in discoloration during storage, as well as an inhibition of lipid oxidation due to the antioxidant content of tomato.

On the other hand, nitrate (NO_3_) is also present in large quantities in a wide variety of vegetables, such as red beet, ginger, spinach, lettuce, radish, arugula, chard, celery, arugula, pea, broccoli, or cabbage [123], and can therefore also be used to replace synthetic additives in meat products. However, the nitrate content in these vegetables can vary significantly between batches, which may affect their consistency and efficacy in inhibiting pathogenic bacteria such as *Clostridium botulinum*. Martínez-Zamora et al. [124] observed that a combination of citrus and natural sources of nitrates (beet, lettuce, arugula, spinach, celery, chard, and watercress) showed synergistic behavior and allowed the maintenance of spanish chorizo samples for 150 days in refrigerated storage without modifying their sensory quality. Another study reported that parsley extract can be used as a substitute for nitrite in mortadella-type sausages without negatively affecting the sensory characteristics of the final product [125]. Parsley extract inhibited the growth of *L. monocytogenes*, thus increasing the shelf life of the product during storage.

Another additive used as a preservative in meat products are sulfites (SO_3_), which are used to maintain color and prolong shelf life [126]. However, their main function is to act as an antibacterial agent against gram-negative bacteria. Despite their technological qualities, these additives when inadequately metabolized, can cause harmful reactions and act as allergens, especially towards asthmatic people, including triggering anaphylactic reactions, hypotension, or dermatitis [127]. For these reasons, the use of sulfites in meat products is regulated by the EU in the form of food additives (E-220 to E-228). Moreover, their use is limited to a few products such as salsicha fresca, longaniza fresca, butifarra fresca, breakfast sausages, and burger meat (with a minimum vegetable and/or cereal content of 4% mixed within the meat) and up to a maximum dose of 450 mg/kg of meat. Bellés et al. [128] observed the effect of carvacrol on lamb hamburgers, obtaining as a result that it could be a good substitute for sulfites, since the reformulated hamburgers obtained lower microbial counts after the use of this essential oil. Some authors have studied that essential oils could act as preservatives in meat products [128,129], this effect being due mainly to the high content of phenolic compounds present in these compounds.

### 4.3. Substitution of Phosphates

Phosphoric acid and phosphates (E-338–341; E-343) and polyphosphates (E-450–452) are compounds commonly used in processed meats as food additives. Their divalent cation chelating capacity and high ionic strength increase the WHC of meat, improving its texture and sensory qualities [130]. Importantly, their primary technological role is related to their interaction with the actomyosin complex, which enhances protein solubility and water retention. On the other hand, phosphates also provide antimicrobial qualities in meat, in addition to inhibiting lipid oxidation, which conditions the color and flavor of the products.

**Figure 2:**
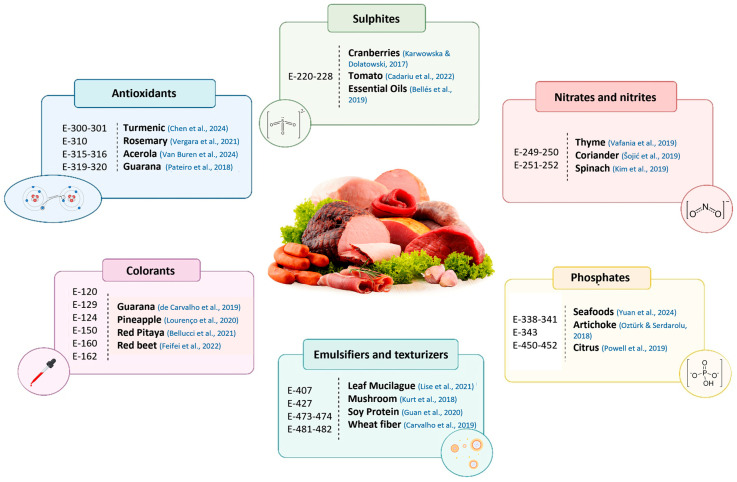
Application of natural extracts in the replacement of E-number food additives in meat products. Data compiled from previously published studies [43,93,97,100,101,115,117,118,120,122,128,133,134,135,136,137,138,139,140,141,142].

Despite its technological benefits, phosphate consumption is risky for people with chronic kidney disease, as its excess in blood is associated with cardiovascular risk [131]. Furthermore, although for healthy individuals phosphates present no concern with respect to carcinogenicity and their acute oral toxicity is low, the EFSA found that exposure was higher than the acceptable daily intake for some population groups [132]. For these reasons, it is necessary to reduce the use of phosphates in the meat industry, seeking other natural alternatives that do not compromise the functions provided by these additives (Figure 2).

Recent research has studied the use of different fibers and vegetable extracts as substitutes for phosphates in meat. Vegetable fibers present potential as functional alternatives to phosphates due to their technological advantages such as high water and oil retention capacity, as well as greater emulsion stability and improved texture [130]. Fibers of plant origin can bind a lot of water without the need for heat and are becoming interesting options to replace phosphates. For example, multiple functional ingredients can be obtained from plums, including substances such as pectin and sorbitol, which are effective in retaining moisture. Marine-derived fibers have also been used as phosphate substitutes. Yuan et al. [133] studied the effect of dietary fiber from seafoods in the reformulation of frankfurters. In this study, it was observed that a 1% reformulation with dietary fiber could completely replace the function of phosphate; however, the incorporation of seafood dietary fiber potentially influenced the aroma and flavor of frankfurters. Jerusalem artichoke powder, alone or in combination with sodium carbonate, can also be used as a substitute for sodium tripolyphosphate (STPP) [134]. The results of a study conducted with emulsified chicken meatballs showed that Jerusalem artichoke exhibited favorable technological properties in terms of water–oil binding and gelling, providing higher water holding capacity and higher emulsion stability of the meatballs than control samples with STPP.

Another compound capable of substituting the function of phosphates due to its high water holding capacity is fructooligosaccharides (FOS). Öztürk-Kerimoğlu & Serdaroğlu [143] studied the effect of inulin as a phosphate substitute in chicken fillets, observing an increase in protein solubility and an achievement of pH and sensory characteristics equivalent to phosphate-containing samples. Some by-products from the food industry can also be used to substitute the functions of these additives; good water and oil retention capacity has been observed in broccoli, apple artichoke, and carob by-products [144], mainly due to their high soluble fiber and total fiber content. The revalorization of nutrients from biowaste can be an interesting alternative to avoid waste in the food industry and at the same time produce healthier products. In a study carried out in Bologna sausages, citrus fiber, a by-product of the juice industry, was used in different concentrations to replace tripolyphosphates [135]. In this study, acceptable technological parameters were achieved, obtaining similar cooking/cooling yields and emulsion stability compared to the sodium tripolyphosphate control.

On the other hand, the use of more texturizing and emulsifying additives is permitted in meat products, such as carrageenans (E-407), cassia gum (E-427), sucrose esters of fatty acids (sucroglycerides) (E-473-474), or stearoyl-2-lactylates (E-481-482). However, the health effects of some of these additives are in dispute and some research has shown that they may have negative gastrointestinal effects [132,145]. Recent research has shown that certain extracts from wheat fiber [136], leaf mucilage [137], mushrooms [138], or soy protein [139] could act as emulsifiers or texturizers in various meat products, improving their appearance and commercial acceptability.

### 4.4. Substitution of Food Colorants

Color is a fundamental sensory attribute that determines consumer perceptions of the acceptability and desirability of meat products, playing a fundamental role in the perception of freshness, quality and palatability of the product [146]. For this reason, the use of food colorants is fundamental in the meat industry to improve the appearance of food products that could be affected by degradation, as well as to color products that would otherwise be colorless. The use of colorants in meat products is limited and regulated in the form of additives by the EU, only a few are accepted and most of them are limited to some specific doses and products.

Only two synthetic colorants are authorized in specific meat products: Ponceau 4R (E-124), permitted only in chorizo and salchichón; and Allura Red AC (E-129), allowed in breakfast sausages with ≥6% cereal content, burger meat with ≥4% vegetable/cereal content, and luncheon meat. However, the use of synthetic colorants has alerted national safety agencies due to their potential toxicity to humans. It has been shown that these colorants can generate carcinogenic metabolites and cause hypersensitivity reactions, leading to health problems [147]. On the other hand, there are additives of natural origin permitted in meat products, such as carminic acid (E-120), caramels (E-150), carotenes (E-160), capsanthin (E-160), and betaines (E-162). However, although these colorants are permitted and natural, they present some limitations, such as instability under certain pH or light conditions, poor fat solubility, or limited thermal resistance. In addition, it is necessary to point out that all these colorants are labeled as E-numbers, which leads to great rejection by the consumer, making it necessary to study new natural colorants in meat products. Several fruits and plants can be used as natural meat colorants due to their pigments and antioxidant properties. Extracts from guarana [140], pineapple [141], jabuticaba [148], and different berries [149] have been identified as natural sources of color alternative to the use of additives, providing attractive reddish colors to meat products. Feifei et al. [142] studied red yeast rice and red beet as colorants in chicken sausages, obtaining good results in color, cooking loss, texture, and sensory evaluation. Finally, the functions of red pitaya extracts were evaluated in pork patties. These extracts correctly simulated the reddish color of pork patties, obtaining as a conclusion that they could be used as a natural colorant to improve the consumer acceptance in concentrations equal to or greater than 0.1 [43].

## 5. Incorporation of Dietary Fiber

Dietary fiber (DF) is defined as the edible parts of plants that can be partially or completely fermented in the large intestine and not digested or absorbed in the human small intestine [150]. Dietary fiber can be found in a large number of fruits, vegetables, and cereals, such as oats, artichoke, peas, sweet potato, lentils, carob, pears, broccoli, apple, etc. [151]. Depending on its solubility, DF can be divided into insoluble dietary fiber (IDF) and soluble dietary fiber (SDF). SDF is composed of non-cellulosic polysaccharides, such as pectins, pentosans, β-glucans, gums, mucilages, and various types of non-digestible oligosaccharides, together with inulin. On the other hand, IDF constitutes the major part of dietary fiber, and includes lignin, cellulose, and hemicelluloses. It has been shown that dietary fiber intake in the diet can provide many health benefits. High dietary fiber intake is associated with reduced premature mortality rates in patients with preexisting cardiovascular disease and hypertension by lowering total cholesterol and low-density lipoprotein cholesterol and reducing systolic and diastolic blood pressure [152].

Further, due to its structure and slow digestion, fiber intake provides a feeling of a full stomach, favoring the feeling of satiety [153]. In addition, fiber reduces transit times, which dilutes and binds toxic chemicals in the colon and reduces mucosal exposure to many of these substances. In addition, dietary fiber ferments in the large intestine with the help of colonic microflora to produce various short-chain fatty acids (SCFAs) that have beneficial health effects, such as acetate, butyrate, and propionate. Many studies have linked these compounds to anti-inflammatory, immunoregulatory, anti-obesity, anti-diabetic, and anti-cancer effects [154]. It has been shown that propionate is able to reduce blood cholesterol levels by inhibiting cholesterol synthesis in liver cells. In addition, many studies have been able to associate butyrate with an inhibition of proliferation, induction of apoptosis, or differentiation of colorectal tumor cells [155].

Due to the numerous benefits of DF, there is increasing interest in its possible addition to reformulated meat products, with the aim of making them healthier. On the other hand, the chemical composition and structure of the fibers, as well as the ionic strength and particle size, greatly influence the water holding capacity and oil-binding capacity of dietary fibers. For this reason, in addition to its nutritional benefits, DF can enhance emulsion stability, texture characteristics, and WHC of meat products. Different fibers used in the formulation of meat products are described in Table 3.

### 5.1. Sensory and Textural Effects of Dietary Fiber in Meat Products

The addition of different sources of fiber is capable of positively or negatively modifying the physicochemical and rheological characteristics of meat products, affecting their sensory characteristics. The water and oil retention properties of SDF can positively affect cooking yield parameters and emulsion stability. These are important quality attributes of meat products, as they are directly linked to reduced production costs by increasing overall output. Mehta et al. [156] evaluated the physicochemical properties, proximate composition, and sensory attributes of chicken patties with added psyllium husk, observing a marked improvement in emulsion stability and cooking yield after reformulation with 4, 6, and 8% psyllium husk. Further, another study examined the effect of three different commercial citrus fibers on spiced beef at a ratio of 0.3–1.0% [166]. In this study, a higher water holding capacity was observed in the patties with citrus fiber, and the highest yield (85.14%) was obtained by adding 0.6% fiber. Finally, Singh et al. [165] evaluated the effect of fiber in chicken meat cutlets reformulated with 2, 4, and 6% carrot powder. The addition of fiber produced a significant improvement in cooking yield and water activity due to the high WHC of carrot powder.

Some fibers are able to change the color and texture of meat products. Recently, the effect of buckwheat fiber on the quality of frankfurters was investigated [158]. In this study, it was found that the addition of buckwheat fiber was able to change the color parameters, increasing the color tonality (h*) and decreasing L* and b* (yellowness) values, negatively affecting consumer acceptability. In addition, the incorporation of this fiber also affected texture parameters, increasing the hardness of the sausages after two weeks of storage. However, various types of fibers have been able to contribute color to meat products. Pumpkin fiber (2%) has been used as added fiber in frankfurters, obtaining as a result a reduction of the lightness and redness scores. Conversely, yellowness score was higher in the frankfurters with added pumpkin [170]. Tomato peel extract has also been used in minced meat at concentrations of 1.5%, 3%, and 4.5%, resulting in very acceptable acceptability for the final product [159].

On the other hand, it has been seen that the use of fibers at low concentrations may not affect the technological properties of meat products. Powell et al. [167] observed the effect of low concentrations of citrus fiber (0.25 and 0.5%) in deli-style turkey breast. This study resulted in the maintenance of sensory qualities (hardness, resilience, cohesiveness, springiness, and chewiness) and color (a*, b*, and L*) of the reformulated product.

### 5.2. Addition of Dietary Fiber to Meat Products to Extend Shelf Life

One of the most important challenges in the formulation of meat products is to inhibit or delay their capacity for lipid and protein oxidation, as well as to avoid microbial growth. It has been reported that dietary fiber, especially SDF, can have a greater antioxidant capacity due to its capacity to bind phenolic compounds [144]. Tarasevičienė et al. [161] studied the ability of raspberry and blackberry pomace (1, 3, and 5%) to inhibit lipid oxidation and prolong the refrigerated storage of beef patties. These two extracts proved to be very effective in relation to lipid oxidation after storage for 9 days at 4 °C t, as only 1% berry pomace influenced the decrease of TBARS values in patties stored for nine days by 3.06-fold. The inclusion of chia seeds in camel burger was also found to have recorded a reduction in TBARS values. In addition, these patties were found to be organoleptically acceptable after refrigerated storage for 12 days [169]. D’Ambra et al. [160] evaluated the effect of adding 2.5% hazelnut peel and dried tomato peel to pork patties against oxidation events. After microbiological, sensory, and physicochemical analyses during 7 days of refrigerated storage (0–4 °C), it was found that the hazelnut skin exerted protection against lipid oxidation, without affecting the sensory quality. However, tomato skin did not exert this protective effect against oxidation. On the other hand, the effect of psyllium addition has been evaluated as a fiber addition and fat replacement in salami [157]. This study replaced fat by 15 and 30% using a hydrogel containing 10% psyllium fiber, 4% carrageenan, and 86% water. Replacing fat with psyllium significantly reduced the oxidation of salami after 21 and 28 days of storage. In addition, the 15% reformulation produced no sensory effects.

Additionally, many fibers have the capacity to prevent microbial growth, which can be useful in the preservation of meat products. This is due to the content of flavonoids such as catechin, epicatechin, procyanidins, and other chemicals with antimicrobial properties, which may include alteration of the cytoplasmic membrane and blocking of specific metabolic pathways and enzymes, leading to DNA damage or inhibition of nucleic acid synthesis by bacteria cells [171]. Babaoğlu et al. [162] evaluated the effect of different berry pomace extracts on beef patties. In addition to finding a positive effect on lipid and protein oxidation protection, beef patties that included berry pomace extracts had lower coliform bacteria counts than the control patty without extract (*p* < 0.05) after 9 days of refrigerated storage. These results suggest that blackberry pomace extract may be a promising natural preservative for processed meat products.

### 5.3. Health Benefits Derived from the Incorporation of Fiber in Meat Products

In addition to the aforementioned beneficial health effects of dietary fiber, some studies have been able to study whether its incorporation in the form of a functional ingredient can modify the negative health attributes associated with meat. Recent studies have shown that meat intake, particularly processed red meat, can modify gut microbiota composition, functional capacity, and health-related biomarkers [172]. For this reason, different studies have tested if the incorporation of fiber-rich extracts in meat products can positively alter the gut microbiota. Thøgersen et al. [173] studied how the addition of inulin in frankfurters might affect the gut microbiota of rats. This study resulted in a remarkable change in fecal microbial composition, measured by 16S rRNA gene amplicon sequencing. On the other hand, rats fed inulin-enriched sausages obtained higher SCFA levels in the fecal and plasma metabolome and higher fecal levels of *Bifidobacterium* spp. compared to rats fed non-enriched sausages. A subsequent study by these investigators was able to reduce fecal concentrations of apparent total N-nitroso compounds (ATNCs) (*p* = 0.03) and nitrosylated iron compounds (FeNO) (*p* = 0.04) following the consumption of inulin-enriched sausage for 4 weeks by healthy Sprague-Dawley rats [174].

Another study evaluated the effect of salami supplemented with 2% of different types of fibers (citrus fiber, arabinogalactan, and inulin) [168]. After an in vitro batch fermentation, it was observed that the addition of any type of dietary fiber to the salami formulation increased the antioxidant capacity of the salami and the amount of SCFAs produced during microbiota fermentation, especially acetic acid. These effects were greater for salami with citrus fiber. A continuation of this study in a 4-week nutritional intervention model [175], with placebo and two parallel groups showed that salami enriched with citrus fiber could increase butyric acid production, positively altering gut microbiota diversity. In addition, the group which consumed reformulated salami reported a decrease in the inflammatory markers TNF-α and CRP due to the incorporation of dietary fiber. Incorporating bioactive compounds into meat products, such as salami, can improve their health benefits by enhancing antioxidant capacity, supporting a healthier gut microbiota, increasing the production of beneficial short-chain fatty acids, and reducing inflammation markers. This suggests that functional meat products can contribute positively to overall health when properly formulated [175,176,177,178].

Ayuso et al. [163] observed the effect of the addition of 3% fiber in additive- and allergen-free cooked ham and turkey breast. These authors observed significant changes in various bacterial species such as *Collinsella aerofaciens* and *Butyricimonas paravirosa* after in vitro batch fermentation. In addition, the introduction of inulin increased the production of different SCFAs, such as acetic and propionic acid. Fernández et al. [164] examined the potential of the reformulation of two meat products reformulated with inulin for the protection of colorectal cancer in an animal model. In this study, chorizo sausages and cooked ham were reformulated with inulin (15.7% and 10%, respectively) and their effect was tested in rats, showing a statistically significant reduction of 49% in the number of colon polyps in the functional meat product cohorts with respect to the control meat product animals, as well as an increase of 51.8% in the production of propionate in the colon. In addition, there was an increase in Bacteroidetes populations after the consumption of functional meat products, and a reduction in Firmicutes, which is associated with the intake of prebiotics.

## 6. Conclusions and Future Perspectives

The interest in healthier meat products has been growing in recent years among consumers and the food industry. The growing criticism of high meat consumption due to its elevated saturated fat content, absence of dietary fiber, and the use of additives in their preservation has increased interest in the nutritional reformulation of these products by means of innovative approaches. The use of hydrogels and oleogels has become an alternative capable of maintaining the technological qualities of meat while introducing healthier oils. On the other hand, the use of extracts from fruits, vegetables, and herbs is an attractive option to replace certain synthetic additives whose toxicity in the human organism is in dispute.

Furthermore, nutritional reformulation should not only aim to reduce harmful components but also promote the development of a wider range of functional and therapeutic meat products. This would allow consumers to choose products that are better adjusted to their health needs or specific nutritional objectives. However, more research is needed on new technologies and extracts that can be used in meat reformulation, as only a relatively limited number of them are capable of improving or maintaining the sensory quality of traditional meat products. In addition, many of the bioactive compound extraction technologies employed require the use of large amounts of harmful solvents, increased time and energy consumption, or hazardous effects on the environment. For this reason, it is also necessary to involve new green technologies in obtaining useful extracts for reformulation, such as the application of pulsed electrical fields (PEFs) or ultrasound-assisted extraction (UAE), among others. Finally, it is necessary to show the real benefits of the nutritional reformulation of meat, especially those with functional or therapeutic purposes, with its verification in experiments with animal models or interventional trials involving humans being necessary in order to demonstrate the real and sustained applicability over time of healthier meat products.

## Figures and Tables

**Table 1:** Fat substitution/enrichment strategies for the reformulation of meat products.

	Fat Substitute	Meat Product	Replacement	Outcomes	Ref.
Microencapsulation	Microencapsulated fish oil	Poultry sausage batters	30%	Reduction in pH and water activity value in the meat batter	[20]
				Improved elastic properties and dynamic viscosity	
	Chia microparticles enriched with rosemary	Beef patties	50%	Decrease in volatiles from lipid and protein oxidation and an increase in terpenes at the beginning and at the end of storage	[21]
				Positive sensory scores	
	Microencapsulated tiger nut, chia, and linseed oils	Deer pâté	50 and 100%	Modification of color parameters, especially tiger nut oil	[22]
				Decreasing the total amount of SFAs and increasing PUFAs of chia and linseed pâtés	
				Higher TBARS values due to vegetable oils incorporation	
Interstification	Milkfat-rapeseed oil	Meat batters	100%	Higher content of unsaturated fatty acids in meat batters	[23]
				Significantly lower apparent viscosity of reformulated meat batter	
	Palm kernel oil	Beef frankfurters	25, 50, 75 and 100%	75% and 100% beef fat replacement significantly lower hardness values	[24]
				Higher TBARS values in frankfurters manufactured with kernel oil	
Emulsion gel	Grape seed oil	Pork emulsions	50%	Increase in moisture, lightness, viscosity and emulsion stability	[25]
				Decreased TBARS values	
	Chia oil	Bologna-type sausages	100%	Higher levels of PUFAs in treatments with emulsion gel	[26]
				Similar purchase intention, taste, color and aroma	
	Tiger nut oil	Beef patties	50 and 100%	Inhibition of lipid oxidation after reformulation of 50 and 100% of fat	[27]
				Decrease in SFAs and enhancement of unsaturated fatty acids	
	Corn, grape seed, soybean, olive, and coconut oil	Duck meat emulsion	100%	Improvement of cooking loss and emulsion stability	[28]
				Decrease of TBARS values in reformulated emulsions	
Oleogel	Linseed oil	Pork pâté	60 and 100%	No impact in mechanical, thermal, or rheological properties	[29]
				Improved the oxidative stability of the organogels and pâtés in partially or totally replace pork backfat	
	Olive, linseed, and fish oil mixture	Pork burgers	100%	Improved PUFA/SFA ratio	[30]
				Softer texture and no important changes in optical properties	
	Sunflower oil	Bologna-type sausages	25, 50, 75 and 100%	Increase of oleic acid and decrease of linoleic acid	[31]
				Emulsion stability increased and cooking loss decreased with increasing the pork back fat replacement by oleogel	
				No effect of the sensory quality up to 50% fat replacement	

PUFA: polyunsaturated fatty acid; SFA: saturated fatty acid; TBARS: thiobarbituric acid reactive substance.

**Table 2:** NaCl reduction strategies in meat products.

	Meat Product	Formulation	Outcomes	Ref.
Chloride salts	Pork sausages	Replacement of 2% NaCl with three different formulations (NaCl + KCl, NaCl + Sub4Salt^®^, and KCl + Sub4Salt^®^) in pork sausages	No effect on lipid oxidation (TBARS) due to reformulation	[69]
			No significant differences in sensory and microbiological analysis between the different formulations	
			Similar effect of odor intensity, color, and bitterness, but less saltiness, in the partially reformulated products	
	Goat patties	Development of low sodium goat patties by applying a mixture of salts consisting of 1% NaCl, 0.4% KCl, and 0.2% CaCl2	Increase in hardness, gumminess, and chewiness of reformulated patties	[70]
			Flavor and saltiness decrease of partially replaced patties compared to control	
	Dry-cured meat sausages	Replacement of the 33% NaCl of dry-cured meat sausage with KCl and aromatic plant extracts	The formulations did not affect the growth of lactic acid bacteria, enterococci, and coagulase-negative staphylococci	[71]
			No changes in color and lipid oxidation parameters between formulations	
			Formualtion with 1% NaCl and 0.5% KCl was considered with the ideal saltiness by 54% consumers	
Seafoods	Beef patties	Substitution of salt by sea spaghetti (0–5%) in beef patties	1% added algae could replace NaCl in patties without deteriorating their appeal among regular consumers	[72]
			Inclusion of >2.5% sea spaghetti resulted in significantly lower overall liking and reduced purchase intention	
	Pork sausages	Incorporation of 2.5% sea spaghetti and Irish wakame as NaCl substitutes in the formulation of low-fat sausages	Darkening of fat and salt-reduced sausages containing seaweed in their formulation	[73]
			High acceptance of sausages with 2.5% sea spaghetti and 0.5% salt and sausages with 2.5% wakame and 1% salt	
	Chicken patties	Addition of seaweed (*Kappaphycus alvarezii*) (2% and 4%) as a salt replacer (1% and 1.5%) in chicken patties	Darker color of chicken patties with seaweeds, lower L*	[74]
			Chicken patties with 2–4% algae showed higher WHC	
			Highest overall acceptability of chicken patties with 1.5% salt and 4% seaweed	
	Turkey sausages	Addition of lyophilized aqueous extract of *Cystoseira barbata* (0.01–0.4%) in turkey meat sausage.	Reduction of lipid oxidation of meat by approximately 36%	[75]
			Improvement of meat color stability during 15 days of refrigeration	
	Pork frankfurters	Salt replacement by 1% of red (*Porphyra umbilicalis* and *Palmaria palmata*) and brown (*Himanthalia elongata* and *Undaria pinnatifida*) edible seaweeds in pork frankfurters	Darker color of pork frankfurters with seaweeds	[76]
			Significant changes in the overall acceptability of reformulated frankfurters, *H. elongata* extract being the most widely accepted	
Fungi	Beef patties	Addition of 2.5 and 5.0% edible mushroom flours (*Agaricus bisporus* and *Pleurotus ostreatus*) during cold storage in beef patties.	Improvement of dietary fiber and protein content with 5% of *A. bisporus* flour	[77]
			Color and flavor modification due to reformulation, but acceptable sensory palatability	
	Beef burgers	Replacement of NaCl with 1.35% of different mushroom flour (*Pleurotus ostreatus*, *Agaricus bisporus*, and *Agaricus brunnescen*) in beef burgers.	Higher lipid oxidation values (0.18–0.20 mg MDA/kg sample) than the control sample	[78]
			No difference in sensory aspects due to reformulation with mushroom flours.	
	Pork frankfurters	Salt reduction in Frankfurter sausages by the addition of 2.5 and 5% *Agaricus bisporus* and *Pleurotus ostreatus* flour	Inhibition of lipid oxidation after reformulation similar to control frankfurters	[79]
			Modification of color and texture, with the *A. bisporus* samples being darker	
Plants and fruits	Pork frankfurters	NaCl replacement with 3 and 6% soy protein isolate in pork frankfurters	Increase in cooking yield, a* value and hardness of reformulated frankfurter sausage	[80]
			Decrease in elasticity and cohesiveness of sausages with 6% soy protein isolate	
	Marinated chicken breast	Addition of 2% seasoning obtained from red grape skins to marinated low-salt chicken breasts	Reformulation with 0.5% salt and 2% grape had the same shelf life as the 2% salt formulation	[81]
			Color of the marinated chicken breasts was less accepted than the control	
	Beef patties	Application of wine pomace (2% *w/w*) as a salt substitute in beef patties with different salt levels	Higher levels of K, Ca, fiber, and phenols in reformulated patties	[82]
			Improvement of the microbial stability of patties, delaying the total growth of mesophilic aerobic and lactic aerobic bacteria	

MDA: malondialdehyde; TBARS: thiobarbituric acid reactive substance; WHC: water holding capacity.

**Table 3:** Different fibers used in the formulation of meat products.

Fiber	Meat Product	Outcomes	Ref.
Psyllium	Chicken patties, salami	Improvement in emulsion stability and cooking yield after reformulation with psyllium husk in chicken patties	[156]
		Reduced oxidation of salami after 21 and 28 days of storage by replacing 15% and 30% of fat with psyllium gel	[157]
Buckwheat husk	Pork Frankfurters	Decrease in L* and b* and increase in hardness of frankfurters	[158]
Tomato pomace	Minced meat, beef burgers	Improved acceptability at concentrations of 1.5%, 3% and 4.5% in minced meat	[159]
		No inhibition of lipid oxidation (TBARS) in beef burgers	[160]
Berry pomace	Beef patties	Inhibition of lipid oxidation (TBARS) in refrigerated storage of beef patties	[161]
		Reduction of coliform bacterial counts after 9 days in beef patties reformulated with berry pomace	[162]
Inulin	Cooked ham, turkey breast, chorizo sausages	Increased production of acetic acid and propionic acid after in vitro fermentation of cooked meat products	[163]
		Reduction of colon cancerous polyps in rats after consumption of chorizo and cooked ham with inulin	[164]
Carrot	Chicken cutlets	Enhancement of cooking yield and water activity after the addition of 2, 4, and 6% of carrot powder	[165]
Citrus fiber	Beef patties, turkey breast, salami	Improvement of water holding capacity and cooking yield in burgers with citrus fiber	[166]
		Maintenance in sensory qualities (hardness, resilience, and chewiness) and color in turkey breast	[167]
		Increase of the antioxidant capacity and the amount of SCFA produced after in vitro fermentation in salami	[168]
Chia seeds	Camel patties	Inhibition of lipid oxidation (TBARS) in refrigerated storage of camel patties with good sensory acceptability	[169]

SCFA: short-chain fatty acids; TBARS: thiobarbituric acid-reactive substances.

## Data Availability

No new data were created or analyzed in this study. Data sharing is not applicable to this article.

## References

[1] Leroy F., Smith N.W., Adesogan A.T., Beal T., Iannotti L., Moughan P.J., Mann N. (2023). The Role of Meat in the Human Diet: Evolutionary Aspects and Nutritional Value. Anim. Front..

[2] Foster D., Haubrick K. (2024). Considering the Impact of Elevated Saturated Fat Intake in the Context of Full Fat Dairy Products and Red Meats on Cardiovascular Health, A Systematic Review. Res. Rev..

[3] Johnston B., De Smet S., Leroy F., Mente A., Stanton A. (2023). Non-Communicable Disease Risk Associated with Red and Processed Meat Consumption—Magnitude, Certainty, and Contextuality of Risk?. Anim. Front..

[4] Zhou D.D., Luo M., Shang A., Mao Q.Q., Li B.Y., Gan R.Y., Li H. (2021). Bin Antioxidant Food Components for the Prevention and Treatment of Cardiovascular Diseases: Effects, Mechanisms, and Clinical Studies. Oxid. Med. Cell Longev..

[5] Badar I.H., Liu H., Chen Q., Xia X., Kong B. (2021). Future Trends of Processed Meat Products Concerning Perceived Healthiness: A Review. Compr. Rev. Food Sci. Food Saf..

[6] Juárez M., Lam S., Bohrer B.M., Dugan M.E.R., Vahmani P., Aalhus J., Juárez A., López-Campos O., Prieto N., Segura J. (2021). Enhancing the Nutritional Value of Red Meat through Genetic and Feeding Strategies. Foods.

[7] Young J.F., Skrivergaard S., Therkildsen M., Rasmussen M.K. (2024). Cultured Meat Production—Scale and Quality. Cell Rep. Sustain..

[8] Grigioni M., Pordomingo A., Arturo Domínguez Vara I., Morales Almaráz E., Hong X., Li C., Wang L., Wang M., Grasso S., Monahan F.J. (2023). Consumer Preferences for Processed Meat Reformulation Strategies: A Prototype for Sensory Evaluation Combined with a Choice-Based Conjoint Experiment. Agriculture.

[9] U.S. Department of Health and Human Services, U.S. Department of Agriculture (2020). Dietary Guidelines for Americans, 2020–2025.

[10] Jeong H.Y., Moon Y.S., Cho K.K. (2024). ω-6 and ω-3 Polyunsaturated Fatty Acids: Inflammation, Obesity and Foods of Animal Resources. Food Sci. Anim. Resour..

[11] Barros J.C., Munekata P.E.S., de Carvalho F.A.L., Domínguez R., Trindade M.A., Pateiro M., Lorenzo J.M. (2021). Healthy Beef Burgers: Effect of Animal Fat Replacement by Algal and Wheat Germ Oil Emulsions. Meat Sci..

[12] Astrup A., Magkos F., Bier D.M., Brenna J.T., de Oliveira Otto M.C., Hill J.O., King J.C., Mente A., Ordovas J.M., Volek J.S. (2020). Saturated Fats and Health: A Reassessment and Proposal for Food-Based Recommendations: JACC State-of-the-Art Review. J. Am. Coll. Cardiol..

[13] Jayedi A., Soltani S., Emadi A., Ghods K., Shab-Bidar S. (2023). Dietary Intake, Biomarkers and Supplementation of Fatty Acids and Risk of Coronary Events: A Systematic Review and Dose-Response Meta-Analysis of Randomized Controlled Trials and Prospective Observational Studies. Crit. Rev. Food Sci. Nutr..

[14] Meyer N.M.T., Pohrt A., Wernicke C., Pletsch-Borba L., Apostolopoulou K., Haberbosch L., Machann J., Pfeiffer A.F.H., Spranger J., Mai K. (2024). Improvement in Visceral Adipose Tissue and LDL Cholesterol by High PUFA Intake: 1-Year Results of the NutriAct Trial. Nutrients.

[15] Hooper L., Martin N., Jimoh O.F., Kirk C., Foster E., Abdelhamid A.S. (2020). Reduction in Saturated Fat Intake for Cardiovascular Disease. Cochrane Database Syst. Rev..

[16] Shen J., Liu Y., Wang X., Bai J., Lin L., Luo F., Zhong H. (2023). A Comprehensive Review of Health-Benefiting Components in Rapeseed Oil. Nutrients.

[17] Nieto G., Lorenzo J.M. (2021). Use of Olive Oil as Fat Replacer in Meat Emulsions. Curr. Opin. Food Sci..

[18] Domínguez R., Lorenzo J.M., Pateiro M., Munekata P.E.S., Alves dos Santos B., Basso Pinton M., Cichoski A.J., Bastianello Campagnol P.C. (2024). Main Animal Fat Replacers for the Manufacture of Healthy Processed Meat Products. Crit. Rev. Food Sci. Nutr..

[19] López-Pedrouso M., Lorenzo J.M., Gullón B., Campagnol P.C.B., Franco D. (2021). Novel Strategy for Developing Healthy Meat Products Replacing Saturated Fat with Oleogels. Curr. Opin. Food Sci..

[20] Kawecki K., Rezler R., Baranowska H.M., Stangierski J. (2021). Influence of Fish Oil and Microencapsulated Fish Oil Additives on Water Binding and the Rheological Properties of Poultry Sausage Batters. J. Sci. Food Agric..

[21] Heck R.T., Fagundes M.B., Cichoski A.J., de Menezes C.R., Barin J.S., Lorenzo J.M., Wagner R., Campagnol P.C.B. (2019). Volatile Compounds and Sensory Profile of Burgers with 50% Fat Replacement by Microparticles of Chia Oil Enriched with Rosemary. Meat Sci..

[22] Vargas-Ramella M., Pateiro M., Barba F.J., Franco D., Campagnol P.C.B., Munekata P.E.S., Tomasevic I., Domínguez R., Lorenzo J.M. (2020). Microencapsulation of Healthier Oils to Enhance the Physicochemical and Nutritional Properties of Deer Pâté. LWT.

[23] Wirkowska-Wojdyła M., Chmiel M., Ostrowska-Ligęza E., Górska A., Bryśbryś J., Słowí Nski M., Czerniszewska A. (2020). The Influence of Interesterification on the Thermal and Technological Properties of Milkfat-Rapeseed Oil Mixture and Its Potential Use in Incorporation of Model Meat Batters. Appl. Sci..

[24] Kılıç B., Özer C.O. (2019). Potential Use of Interesterified Palm Kernel Oil to Replace Animal Fat in Frankfurters. Meat Sci..

[25] Kim T.K., Yong H.I., Jung S., Kim Y.B., Choi Y.S. (2020). Effects of Replacing Pork Fat with Grape Seed Oil and Gelatine/Alginate for Meat Emulsions. Meat Sci..

[26] Câmara F.I., Midori Ozaki M., Santos M., Silva Vidal V.A., Oliveira Ribeiro W., de Souza Paglarini C., Bernardinelli O.D., Sabadini E., Rodrigues Pollonio M.A. (2021). Olive Oil-Based Emulsion Gels Containing Chia (*Salvia Hispanica* L.) Mucilage Delivering Healthy Claims to Low-Saturated Fat Bologna Sausages. Food Struct..

[27] Barros J.C., Munekata P.E.S., De Carvalho F.A.L., Pateiro M., Barba F.J., Domínguez R., Trindade M.A., Lorenzo J.M. (2020). Use of Tiger Nut (Cyperus Esculentus L.) Oil Emulsion as Animal Fat Replacement in Beef Burgers. Foods.

[28] Kim T.K., Lee M.H., Kim S.M., Kim M.J., Jung S., Yong H.I., Choi Y.S. (2021). Physiochemical Properties of Reduced-Fat Duck Meat Emulsion Systems: Effects of Preemulsification with Vegetable Oils and Duck Skin. Poult. Sci..

[29] Ramírez-carrasco P., Paredes-toledo J., Romero-hasler P., Soto-bustamante E., Díaz-calderón P., Robert P., Giménez B. (2020). Effect of Adding Curcumin on the Properties of Linseed Oil Organogels Used as Fat Replacers in Pâtés. Antioxidants.

[30] Gómez-Estaca J., Pintado T., Jiménez-Colmenero F., Cofrades S. (2019). Assessment of a Healthy Oil Combination Structured in Ethyl Cellulose and Beeswax Oleogels as Animal Fat Replacers in Low-Fat, PUFA-Enriched Pork Burgers. Food Bioprocess Technol..

[31] da Silva S.L., Amaral J.T., Ribeiro M., Sebastião E.E., Vargas C., de Lima Franzen F., Schneider G., Lorenzo J.M., Fries L.L.M., Cichoski A.J. (2019). Fat Replacement by Oleogel Rich in Oleic Acid and Its Impact on the Technological, Nutritional, Oxidative, and Sensory Properties of Bologna-Type Sausages. Meat Sci..

[32] Öztürk T., Turhan S. (2020). Physicochemical Properties of Pumpkin (*Cucurbita Pepo* L.) Seed Kernel Flour and Its Utilization in Beef Meatballs as a Fat Replacer and Functional Ingredient. J. Food Process Preserv..

[33] Ding Y., Lin H.W., Lin Y.L., Yang D.J., Yu Y.S., Chen J.W., Wang S.Y., Chen Y.C. (2018). Nutritional Composition in the Chia Seed and Its Processing Properties on Restructured Ham-like Products. J. Food Drug Anal..

[34] Barros J.C., Rodrigues I., Pires M.A., Gonçalves L.A., de Carvalho F.A.L., Trindade M.A. (2019). Healthier Chicken Nuggets Incorporated with Chia (*Salvia Hispanica* L.) Flour and Partial Replacement of Sodium Chloride with Calcium Chloride. Emir. J. Food Agric..

[35] de Oliveira Paula M.M., Silva J.R.G., de Oliveira K.L., Massingue A.A., Ramos E.M., Júnior A.A.B., Silva M.H.L., Silva V.R.O. (2019). Technological and Sensory Characteristics of Hamburgers Added with Chia Seed as Fat Replacer. Ciência Rural..

[36] Antonini E., Torri L., Piochi M., Cabrino G., Meli M.A., De Bellis R. (2020). Nutritional, Antioxidant and Sensory Properties of Functional Beef Burgers Formulated with Chia Seeds and Goji Puree, before and after in Vitro Digestion. Meat Sci..

[37] Vargas-Ramella M., Munekata P.E.S., Gagaoua M., Franco D., Campagnol P.C.B., Pateiro M., Barretto A.C.d.S., Domínguez R., Lorenzo J.M. (2020). Inclusion of Healthy Oils for Improving the Nutritional Characteristics of Dry-Fermented Deer Sausage. Foods.

[38] Heck R.T., Lorenzo J.M., Dos Santos B.A., Cichoski A.J., de Menezes C.R., Campagnol P.C.B. (2021). Microencapsulation of Healthier Oils: An Efficient Strategy to Improve the Lipid Profile of Meat Products. Curr. Opin. Food Sci..

[39] Zhu Y., Chen X., McClements D.J., Zou L., Liu W. (2018). PH-, Ion- and Temperature-Dependent Emulsion Gels: Fabricated by Addition of Whey Protein to Gliadin-Nanoparticle Coated Lipid Droplets. Food Hydrocoll..

[40] Ren Y., Huang L., Zhang Y., Li H., Zhao D., Cao J., Liu X., Ren Y., Huang L., Zhang Y. (2022). Application of Emulsion Gels as Fat Substitutes in Meat Products. Foods.

[41] Lin D., Kelly A.L., Miao S. (2020). Preparation, Structure-Property Relationships and Applications of Different Emulsion Gels: Bulk Emulsion Gels, Emulsion Gel Particles, and Fluid Emulsion Gels. Trends Food Sci. Technol..

[42] Paglarini C.d.S., Vidal V.A.S., Martini S., Cunha R.L., Pollonio M.A.R. (2022). Protein-Based Hydrogelled Emulsions and Their Application as Fat Replacers in Meat Products: A Review. Crit. Rev. Food Sci. Nutr..

[43] Bellucci E.R.B., Munekata P.E.S., Pateiro M., Lorenzo J.M., da Silva Barretto A.C. (2021). Red Pitaya Extract as Natural Antioxidant in Pork Patties with Total Replacement of Animal Fat. Meat Sci..

[44] Domínguez R., Bohrer B., Munekata P.E.S., Pateiro M., Lorenzo J.M. (2021). Recent Discoveries in the Field of Lipid Bio-Based Ingredients for Meat Processing. Molecules.

[45] Pintado T., Herrero A.M., Jiménez-Colmenero F., Pasqualin Cavalheiro C., Ruiz-Capillas C. (2018). Chia and Oat Emulsion Gels as New Animal Fat Replacers and Healthy Bioactive Sources in Fresh Sausage Formulation. Meat Sci..

[46] Lucas-González R., Roldán-Verdu A., Sayas-Barberá E., Fernández-López J., Pérez-Álvarez J.A., Viuda-Martos M. (2020). Assessment of Emulsion Gels Formulated with Chestnut (Castanea Sativa M.) Flour and Chia (*Salvia Hispanica* L) Oil as Partial Fat Replacers in Pork Burger Formulation. J. Sci. Food Agric..

[47] Heck R.T., Saldaña E., Lorenzo J.M., Correa L.P., Fagundes M.B., Cichoski A.J., de Menezes C.R., Wagner R., Campagnol P.C.B. (2019). Hydrogelled Emulsion from Chia and Linseed Oils: A Promising Strategy to Produce Low-Fat Burgers with a Healthier Lipid Profile. Meat Sci..

[48] Alejandre M., Ansorena D., Calvo M.I., Cavero R.Y., Astiasarán I. (2019). Influence of a Gel Emulsion Containing Microalgal Oil and a Blackthorn (*Prunus Spinosa* L.) Branch Extract on the Antioxidant Capacity and Acceptability of Reduced-Fat Beef Patties. Meat Sci..

[49] Öztürk-Kerimoğlu B., Kavuşan H.S., Benzer Gürel D., Çağındı Ö., Serdaroğlu M. (2021). Cold-Set or Hot-Set Emulsion Gels Consisted of a Healthy Oil Blend to Replace Beef Fat in Heat-Treated Fermented Sausages. Meat Sci..

[50] Lima T.L.S., da Costa G.F., da Silva Araújo Í.B., da Cruz G.R.B., Ribeiro N.L., Filho E.M.B., Domínguez R., Lorenzo J.M. (2021). Pre-Emulsioned Linseed Oil as Animal Fat Replacement in Sheep Meat Sausages: Microstructure and Physicochemical Properties. J. Food Process Preserv..

[51] de Souza Paglarini C., Vidal V.A.S., Ribeiro W., Badan Ribeiro A.P., Bernardinelli O.D., Herrero A.M., Ruiz-Capillas C., Sabadini E., Rodrigues Pollonio M.A. (2021). Using Inulin-Based Emulsion Gels as Fat Substitute in Salt Reduced Bologna Sausage. J. Sci. Food Agric..

[52] Pintado T., Muñoz-González I., Salvador M., Ruiz-Capillas C., Herrero A.M. (2021). Phenolic Compounds in Emulsion Gel-Based Delivery Systems Applied as Animal Fat Replacers in Frankfurters: Physico-Chemical, Structural and Microbiological Approach. Food Chem..

[53] Alejandre M., Astiasarán I., Ansorena D., Barbut S. (2019). Using Canola Oil Hydrogels and Organogels to Reduce Saturated Animal Fat in Meat Batters. Food Res. Int..

[54] Manzoor S., Masoodi F.A., Rashid R., Naqash F., Ahmad M. (2022). Oleogels for the Development of Healthy Meat Products: A Review. Appl. Food Res..

[55] Ferrer-González B.M., García-Martínez I., Totosaus A. (2019). Textural Properties, Sensory Acceptance and Fatty Acid Profile of Cooked Meat Batters Employing Pumpkin Seed Paste or Soybean Oil Oleogel as Fat Replacers. Grasas Y Aceites.

[56] Domian E., Brynda-Kopytowska A., Marzec A. (2017). Functional Properties and Oxidative Stability of Flaxseed Oil Microencapsulated by Spray Drying Using Legume Proteins in Combination with Soluble Fiber or Trehalose. Food Bioprocess Technol..

[57] Xie X., Gilbert M., Petley-Ragan L., Auld V.J. (2014). Loss of focal adhesions in glia disrupts both glial and photoreceptor axon migration in the Drosophila visual system. Development.

[58] Nazarewicz S., Kozłowicz K., Kobus Z., Gładyszewska B., Matwijczuk A., Ślusarczyk L., Skrzypek T., Sujka M., Kozłowicz N. (2022). The Use of Ultrasound in Shaping the Properties of Ice Cream with Oleogel Based on Oil Extracted from Tomato Seeds. Appl. Sci..

[59] Li H., Zhang L., Jia Y., Yuan Y., Li H., Cui W., Yu J. (2022). Application of whey protein emulsion gel microparticles as fat replacers in low-fat yogurt: Applicability of vegetable oil as the oil phase. J. Dairy Sci..

[60] Oh I., Lee J.H., Lee H.G., Lee S. (2019). Feasibility of Hydroxypropyl Methylcellulose Oleogel as an Animal Fat Replacer for Meat Patties. Food Res. Int..

[61] Ferro A.C., de Souza Paglarini C., Rodrigues Pollonio M.A., Lopes Cunha R. (2021). Glyceryl Monostearate-Based Oleogels as a New Fat Substitute in Meat Emulsion. Meat Sci..

[62] Shao L., Bi J., Dai R., Li X. (2020). Effects of Fat/Oil Type and Ethylcellulose on the Gel Characteristic of Pork Batter. Food Res. Int..

[63] de Carvalho F.A.L., Munekata P.E.S., Pateiro M., Campagnol P.C.B., Domínguez R., Trindade M.A., Lorenzo J.M. (2020). Effect of Replacing Backfat with Vegetable Oils during the Shelf-Life of Cooked Lamb Sausages. LWT.

[64] Martins A.J., Lorenzo J.M., Franco D., Pateiro M., Domínguez R., Munekata P.E.S., Pastrana L.M., Vicente A.A., Cunha R.L., Cerqueira M.A. (2020). Characterization of Enriched Meat-Based Pâté Manufactured with Oleogels as Fat Substitutes. Gels.

[65] Gómez-Estaca J., Pintado T., Jiménez-Colmenero F., Cofrades S. (2020). The Effect of Household Storage and Cooking Practices on Quality Attributes of Pork Burgers Formulated with PUFA- and Curcumin-Loaded Oleogels as Healthy Fat Substitutes. LWT.

[66] Wong K.M., Corradini M.G., Autio W., Kinchla A.J. (2019). Sodium Reduction Strategies through Use of Meat Extenders (White Button Mushrooms vs. Textured Soy) in Beef Patties. Food Sci. Nutr..

[67] Perez L.M., Fang H.Y., Ashrafi S.A., Burrows B.T., King A.C., Larsen R.J., Sutton B.P., Wilund K.R. (2021). Pilot Study to Reduce Interdialytic Weight Gain by Provision of Low-Sodium, Home-Delivered Meals in Hemodialysis Patients. Hemodial. Int..

[68] Kieneker L.M., Eisenga M.F., Gansevoort R.T., de Boer R.A., Navis G., Dullaart R.P.F., Joosten M.M., Bakker S.J.L. (2018). Association of Low Urinary Sodium Excretion With Increased Risk of Stroke. Mayo Clin. Proc..

[69] Teixeira A., Domínguez R., Ferreira I., Pereira E., Estevinho L., Rodrigues S., Lorenzo J.M. (2021). Effect of NaCl Replacement by Other Salts on the Quality of Bísaro Pork Sausages (PGI Chouriça de Vinhais). Foods.

[70] Nayak N.K., Pathak V. (2022). Development and Quality Assessment of Low-Sodium Functional Chevon Patties. Br. Food J..

[71] Bernardo P., Fernandes M.J., Fernandes M.H., Teixeira M.P., Alfaia C.M., Serrano C., Patarata L., Fraqueza M.J. (2025). Salt Reduction Strategies for Dry Cured Meat Products: The Use of KCl and Microencapsulated Spices and Aromatic Plant Extracts. Meat Sci..

[72] Głuchowski A., Crofton E., Inguglia E.S., O’Sullivan M.G., Kerry J.P., Hamill R.M. (2024). Incorporation of Sea Spaghetti (Himanthalia Elongata) in Low-Salt Beef Patties: Effect on Sensory Profile and Consumer Hedonic and Emotional Response. Foods.

[73] Mohammed H.O., O’Grady M.N., O’Sullivan M.G., Kerry J.P. (2024). The Effect of Reducing Fat and Salt on the Quality and Shelf Life of Pork Sausages Containing Brown Seaweeds (Sea Spaghetti and Irish Wakame). Appl. Sci..

[74] Pindi W., Qin L.W., Sulaiman N.S., Mohd Zaini H., Munsu E., Wahab N.A., Mohd Noor N.Q.I. (2023). Effects of Salt Reduction and the Inclusion of Seaweed (Kappaphycus Alvarezii) on the Physicochemical Properties of Chicken Patties. Appl. Sci..

[75] Sellimi S., Benslima A., Ksouda G., Montero V.B., Hajji M., Nasri M. (2018). Safer and Healthier Reduced Nitrites Turkey Meat Sausages Using Lyophilized Cystoseira Barbata Seaweed Extract. J. Complement. Integr. Med..

[76] Vilar E.G., Ouyang H., O’Sullivan M.G., Kerry J.P., Hamill R.M., O’Grady M.N., Mohammed H.O., Kilcawley K.N. (2020). Effect of Salt Reduction and Inclusion of 1% Edible Seaweeds on the Chemical, Sensory and Volatile Component Profile of Reformulated Frankfurters. Meat Sci..

[77] Cerón-Guevara M.I., Rangel-Vargas E., Lorenzo J.M., Bermúdez R., Pateiro M., Rodriguez J.A., Sanchez-Ortega I., Santos E.M. (2020). Effect of the Addition of Edible Mushroom Flours (Agaricus Bisporus and Pleurotus Ostreatus) on Physicochemical and Sensory Properties of Cold-Stored Beef Patties. J. Food Process Preserv..

[78] Botella-Martínez C., Muñoz-Tebar N., Lucas-González R., Pérez-Álvarez J.A., Fernández-López J., Viuda-Martos M. (2023). Assessment of Chemical, Physico-Chemical and Sensory Properties of Low-Sodium Beef Burgers Formulated with Flours from Different Mushroom Types. Foods.

[79] Cerón-Guevara M.I., Rangel-Vargas E., Lorenzo J.M., Bermúdez R., Pateiro M., Rodríguez J.A., Sánchez-Ortega I., Santos E.M. (2020). Reduction of Salt and Fat in Frankfurter Sausages by Addition of Agaricus Bisporus and Pleurotus Ostreatus Flour. Foods.

[80] Kang Z.L., Zou X.L., Meng L., Li Y. (2021). ping Effects of NaCl and Soy Protein Isolate on the Physicochemical, Water Distribution, and Mobility in Frankfurters. Int. J. Food Sci. Technol..

[81] Ortega-Heras M., Villarroel E., Mateos S., García-Lomillo J., Rovira J., González-Sanjosé M.L. (2020). Application of a Seasoning Obtained from Red Grape Pomace as a Salt Replacer for the Elaboration of Marinated Chicken Breasts: Study of Their Physical-Chemical and Sensory Properties and Microbiological Stability. CyTA J. Food.

[82] García-Lomillo J., González-SanJosé M.a.L., Del Pino-García R., Rivero-Pérez M.a.D., Muñiz-Rodríguez P. (2017). Alternative Natural Seasoning to Improve the Microbial Stability of Low-Salt Beef Patties. Food Chem..

[83] Ponzo V., Pellegrini M., Costelli P., Vázquez-Araújo L., Gayoso L., D’eusebio C., Ghigo E., Bo S. (2021). Strategies for Reducing Salt and Sugar Intakes in Individuals at Increased Cardiometabolic Risk. Nutrients.

[84] Santos Sánchez G., Cotas J., Tavares J.O., Silva R., Pereira L. (2024). Seaweed as a Safe Nutraceutical Food: How to Increase Human Welfare?. Nutraceuticals.

[85] Liu Q., Sun L., Ding Y., Zhuang Y. (2024). Chemical Composition, Health Benefits, Food Processing Effects and Applications of Boletus: A Review. Crit. Rev. Food Sci. Nutr..

[86] Banerjee D.K., Das A.K., Banerjee R., Pateiro M., Nanda P.K., Gadekar Y.P., Biswas S., McClements D.J., Lorenzo J.M. (2020). Application of Enoki Mushroom (Flammulina Velutipes) Stem Wastes as Functional Ingredients in Goat Meat Nuggets. Foods.

[87] Cai T., Hai N., Guo P., Feng Z., Zhang Y., Wang J., Yu Z., Liu H., Ding L. (2024). Characteristics of Umami Taste of Soy Sauce Using Electronic Tongue, Amino Acid Analyzer, and MALDI−TOF MS. Foods.

[88] Román S., Sánchez-Siles L.M., Siegrist M. (2017). The Importance of Food Naturalness for Consumers: Results of a Systematic Review. Trends Food Sci. Technol..

[89] Wu H., Richards M.P., Undeland I. (2022). Lipid Oxidation and Antioxidant Delivery Systems in Muscle Food. Compr. Rev. Food Sci. Food Saf..

[90] Ham J., Lim W., Park S., Bae H., You S., Song G. (2019). Synthetic Phenolic Antioxidant Propyl Gallate Induces Male Infertility through Disruption of Calcium Homeostasis and Mitochondrial Function. Environ. Pollut..

[91] Ren J., Li Z., Li X., Yang L., Bu Z., Wu Y., Li Y., Zhang S., Meng X. (2025). Exploring the Mechanisms of the Antioxidants BHA, BHT, and TBHQ in Hepatotoxicity, Nephrotoxicity, and Neurotoxicity from the Perspective of Network Toxicology. Foods.

[92] Khezerlou A., pouya Akhlaghi A., Alizadeh A.M., Dehghan P., Maleki P. (2022). Alarming Impact of the Excessive Use of Tert-Butylhydroquinone in Food Products: A Narrative Review. Toxicol. Rep..

[93] Chen Y., Zha E., Zhang Z., Zhang J., Wang R., Li J., Sun J. (2024). Effect of Combined Treatment of Curcumin and Sodium Bicarbonate on Quality Characteristics of Refrigerated Beef Meatballs. LWT.

[94] de Carvalho F.A.L., Munekata P.E.S., Lopes de Oliveira A., Pateiro M., Domínguez R., Trindade M.A., Lorenzo J.M. (2020). Turmeric (Curcuma Longa L.) Extract on Oxidative Stability, Physicochemical and Sensory Properties of Fresh Lamb Sausage with Fat Replacement by Tiger Nut (*Cyperus Esculentus* L.) Oil. Food Res. Int..

[95] Ansorena D., Astiasaran I. (2024). Natural Antioxidants (Rosemary and Parsley) in Microwaved Ground Meat Patties: Effects of in Vitro Digestion. J. Sci. Food Agric..

[96] Al-Hijazeen M. (2021). The Combination Effect of Adding Rosemary Extract and Oregano Essential Oil on Ground Chicken Meat Quality. Food Sci. Technol..

[97] Vergara H., Cózar A., Rubio N. (2021). Lamb Meat Burgers Shelf Life: Effect of the Addition of Different Forms of Rosemary (*Rosmarinus Officinalis* L.). CyTA J. Food.

[98] de Paiva G.B., Trindade M.A., Romero J.T., da Silva-Barretto A.C. (2021). Antioxidant Effect of Acerola Fruit Powder, Rosemary and Licorice Extract in Caiman Meat Nuggets Containing Mechanically Separated Caiman Meat. Meat Sci..

[99] Nieto G., Ros G., Castillo J. (2018). Antioxidant and Antimicrobial Properties of Rosemary (*Rosmarinus Officinalis*, L.): A Review. Medicines.

[100] Van Buren J.B., Epperson B., Jepsen S., Heimbuch M., Oliver K., Nasados J., Bass P.D., Colle M.J. (2024). Acerola Cherry and Rosemary Extracts Improve Color and Delay Lipid Oxidation in Previously Frozen Beef. Foods.

[101] Pateiro M., Vargas F.C., Chincha A.A.I.A., Sant’Ana A.S., Strozzi I., Rocchetti G., Barba F.J., Domínguez R., Lucini L., do Amaral Sobral P.J. (2018). Guarana Seed Extracts as a Useful Strategy to Extend the Shelf Life of Pork Patties: UHPLC-ESI/QTOF Phenolic Profile and Impact on Microbial Inactivation, Lipid and Protein Oxidation and Antioxidant Capacity. Food Res. Int..

[102] Martínez-zamora L., Ros G., Nieto G. (2020). Synthetic vs. Natural Hydroxytyrosol for Clean Label Lamb Burgers. Antioxidants.

[103] Pateiro M., Gómez-Salazar J.A., Jaime-Patlán M., Sosa-Morales M.E., Lorenzo J.M. (2021). Plant Extracts Obtained with Green Solvents as Natural Antioxidants in Fresh Meat Products. Antioxidants.

[104] Alirezalu K., Pateiro M., Yaghoubi M., Alirezalu A., Peighambardoust S.H., Lorenzo J.M. (2020). Phytochemical Constituents, Advanced Extraction Technologies and Techno-Functional Properties of Selected Mediterranean Plants for Use in Meat Products. A Comprehensive Review. Trends Food Sci. Technol..

[105] Stoica M. (2019). Overview of Sodium Nitrite as a Multifunctional Meat-Curing Ingredient. Ann. Univ. Dunarea De. Jos Galati Fascicle VI Food Technol..

[106] Nicoletti D., Yin B., Schmidt J., Wunch K., Gieg L., Jenneman G. (2025). Metabolic Stress Induced by Nitrite Enhances Biocide Kill of Sulfate Reducing Bacteria in Oilfield Enrichments. Int. Biodeterior. Biodegrad..

[107] Fraqueza M.J., Laranjo M., Elias M., Patarata L. (2021). Microbiological Hazards Associated with Salt and Nitrite Reduction in Cured Meat Products: Control Strategies Based on Antimicrobial Effect of Natural Ingredients and Protective Microbiota. Curr. Opin. Food Sci..

[108] Stoica M., Antohi V.M., Alexe P., Ivan A.S., Stanciu S., Stoica D., Zlati M.L., Stuparu-Cretu M. (2022). New Strategies for the Total/Partial Replacement of Conventional Sodium Nitrite in Meat Products: A Review. Food Bioprocess Technol..

[109] Karwowska M., Kononiuk A. (2020). Nitrates/Nitrites in Food-Risk for Nitrosative Stress and Benefits. Antioxidants.

[110] Schrenk D., Bignami M., Bodin L., Chipman J.K., del Mazo J., Hogstrand C., Hoogenboom L., Leblanc J.C., Nebbia C.S., Nielsen E. (2023). Risk Assessment of N-Nitrosamines in Food. Efsa J..

[111] Ansari F.A., Ali S.N., Arif H., Khan A.A., Mahmood R. (2017). Acute Oral Dose of Sodium Nitrite Induces Redox Imbalance, DNA Damage, Metabolic and Histological Changes in Rat Intestine. PLoS ONE.

[112] Luo Z., Wang H., Lin S., Liao L., Cai L., Zhang X., Tan Y., Shen M. (2022). Study on the Levels of N-Nitrosamine Compounds and Untargeted Metabolomics in Patients with Colorectal Cancer. Anal. Bioanal. Chem..

[113] Gold S.A., Margulis V. (2023). Carcinogenic Effects of Nitrosodimethylamine (NDMA) Contamination in Ranitidine: Defining the Relationship With Renal Malignancies. JU Open Plus.

[114] Zheng J., Daniel C.R., Hatia R.I., Stuff J., Abdelhakeem A.A., Rashid A., Chun Y.S., Jalal P.K., Kaseb A.O., Li D. (2021). Dietary N-Nitroso Compounds and Risk of Hepatocellular Carcinoma: A USA-Based Study. Hepatology.

[115] Karwowska M., Dolatowski Z.J. (2017). Effect of Acid Whey and Freeze-Dried Cranberries on Lipid Oxidation and Fatty Acid Composition of Nitrite-/Nitrate-Free Fermented Sausage Made from Deer Meat. Asian-Australas. J. Anim. Sci..

[116] Salejda A.M., Nawirska-Olszanska A., Janiewicz U., Krasnowska G. (2017). Effects on Quality Properties of Pork Sausages Enriched with Sea Buckthorn (*Hippophae Rhamnoides* L.). J. Food Qual..

[117] Šojić B., Pavlić B., Ikonić P., Tomović V., Ikonić B., Zeković Z., Kocić-Tanackov S., Jokanović M., Škaljac S., Ivić M. (2019). Coriander Essential Oil as Natural Food Additive Improves Quality and Safety of Cooked Pork Sausages with Different Nitrite Levels. Meat Sci..

[118] Vafania B., Fathi M., Soleimanian-Zad S. (2019). Nanoencapsulation of Thyme Essential Oil in Chitosan-Gelatin Nanofibers by Nozzle-Less Electrospinning and Their Application to Reduce Nitrite in Sausages. Food Bioprod. Process..

[119] Golden M., Wanless B., Glass K. (2019). Comparison of Clean Label Antimicrobials with Nitrite on the Inhibition of Clostridium Perfringens during Extended Cooling of a Model Deli-Style Ham Product.

[120] Kim T.K., Hwang K.E., Lee M.A., Paik H.D., Kim Y.B., Choi Y.S. (2019). Quality Characteristics of Pork Loin Cured with Green Nitrite Source and Some Organic Acids. Meat Sci..

[121] Hong J.H., Lee S.H., Kim H.Y. (2021). Effect of Cherry Tomato Paste for Nitrite Replacement on Emulsion-Type Pork Sausage. J. Korean Soc. Food Sci. Nutr..

[122] Cadariu A.I., Cocan I., Negrea M., Alexa E., Obistioiu D., Hotea I., Radulov I., Poiana M.A. (2022). Exploring the Potential of Tomato Processing Byproduct as a Natural Antioxidant in Reformulated Nitrite-Free Sausages. Sustainability.

[123] Hosseini M.J., Dezhangah S., Esmi F., Gharavi-nakhjavani M.S., Hashempour-baltork F., Mirza Alizadeh A. (2023). A Worldwide Systematic Review, Meta-Analysis and Meta-Regression of Nitrate and Nitrite in Vegetables and Fruits. Ecotoxicol. Environ. Saf..

[124] Martínez-Zamora L., Peñalver R., Ros G., Nieto G. (2021). Substitution of Synthetic Nitrates and Antioxidants by Spices, Fruits and Vegetables in Clean Label Spanish Chorizo. Food Res. Int..

[125] Riel G., Boulaaba A., Popp J., Klein G. (2017). Effects of Parsley Extract Powder as an Alternative for the Direct Addition of Sodium Nitrite in the Production of Mortadella-Type Sausages—Impact on Microbiological, Physicochemical and Sensory Aspects. Meat Sci..

[126] Nieto G., Martínez-Zamora L., Peñalver R., Marín-Iniesta F., Taboada-Rodríguez A., López-Gómez A., Martínez-Hernández G.B. (2023). Applications of Plant Bioactive Compounds as Replacers of Synthetic Additives in the Food Industry. Foods.

[127] D’Amore T., Di Taranto A., Berardi G., Vita V., Marchesani G., Chiaravalle A.E., Iammarino M. (2020). Sulfites in Meat: Occurrence, Activity, Toxicity, Regulation, and Detection. A Comprehensive Review. Compr. Rev. Food Sci. Food Saf..

[128] Bellés M., Alonso V., Roncalés P., Beltrán J.A. (2019). Sulfite-Free Lamb Burger Meat: Antimicrobial and Antioxidant Properties of Green Tea and Carvacrol. J. Sci. Food Agric..

[129] Hernández-Hernández E., Lira-Moreno C.Y., Guerrero-Legarreta I., Wild-Padua G., Di Pierro P., García-Almendárez B.E., Regalado-González C. (2017). Effect of Nanoemulsified and Microencapsulated Mexican Oregano (Lippia Graveolens Kunth) Essential Oil Coatings on Quality of Fresh Pork Meat. J. Food Sci..

[130] Thangavelu K.P., Kerry J.P., Tiwari B.K., McDonnell C.K. (2019). Novel Processing Technologies and Ingredient Strategies for the Reduction of Phosphate Additives in Processed Meat. Trends Food Sci. Technol..

[131] Rubio-Aliaga I., Krapf R. (2022). Phosphate Intake, Hyperphosphatemia, and Kidney Function. Pflügers Arch. Eur. J. Physiol..

[132] Younes M., Aquilina G., Castle L., Engel K.H., Fowler P., Frutos Fernandez M.J., Fürst P., Gürtler R., Husøy T., Mennes W. (2019). Re-Evaluation of Phosphoric Acid–Phosphates—Di-, Tri- and Polyphosphates (E 338–341, E 343, E 450–452) as Food Additives and the Safety of Proposed Extension of Use. EFSA J..

[133] Yuan D., Liang X., Kong B., Xia X., Cao C., Zhang H., Liu Q., Li X. (2024). Influence of Seaweed Dietary Fibre as a Potential Alternative to Phosphates on the Quality Profiles and Flavour Attributes of Frankfurters. Meat Sci..

[134] Oztürk B., Serdarolu M. (2018). Effects of Jerusalem Artichoke Powder and Sodium Carbonate as Phosphate Replacers on the Quality Characteristics of Emulsified Chicken Meatballs. Food Sci. Anim. Resour..

[135] Powell M.J., Sebranek J.G., Prusa K.J., Tarté R. (2019). Evaluation of Citrus Fiber as a Natural Replacer of Sodium Phosphate in Alternatively-Cured All-Pork Bologna Sausage. Meat Sci..

[136] Carvalho L.T., Pires M.A., Baldin J.C., Munekata P.E.S., de Carvalho F.A.L., Rodrigues I., Polizer Y.J., de Mello J.L.M., Lapa-Guimarães J., Trindade M.A. (2019). Partial Replacement of Meat and Fat with Hydrated Wheat Fiber in Beef Burgers Decreases Caloric Value without Reducing the Feeling of Satiety after Consumption. Meat Sci..

[137] Guan H., Diao X., Liu D., Han J., Kong B., Liu D., Gao C., Zhang L. (2020). Effect of High-Pressure Processing Enzymatic Hydrolysates of Soy Protein Isolate on the Emulsifying and Oxidative Stability of Myofibrillar Protein-Prepared Oil-in-Water Emulsions. J. Sci. Food Agric..

[138] Kurt A., Gençcelep H. (2018). Enrichment of Meat Emulsion with Mushroom (Agaricus Bisporus) Powder: Impact on Rheological and Structural Characteristics. J. Food Eng..

[139] Lise C.C., Marques C., da Cunha M.A.A., Mitterer-Daltoé M.L. (2021). Alternative Protein from Pereskia Aculeata Miller Leaf Mucilage: Technological Potential as an Emulsifier and Fat Replacement in Processed Mortadella Meat. Eur. Food Res. Technol..

[140] de Carvalho F.A.L., Lorenzo J.M., Pateiro M., Bermúdez R., Purriños L., Trindade M.A. (2019). Effect of Guarana (Paullinia Cupana) Seed and Pitanga (Eugenia Uniflora L.) Leaf Extracts on Lamb Burgers with Fat Replacement by Chia Oil Emulsion during Shelf Life Storage at 2 °C. Food Res. Int..

[141] Lourenço S.C., Fraqueza M.J., Fernandes M.H., Moldão-Martins M., Alves V.D. (2020). Application of Edible Alginate Films with Pineapple Peel Active Compounds on Beef Meat Preservation. Antioxidants.

[142] Feifei S., Kryzhska T.A., Yan L., Zhenhua D., Danylenko S.H., Stepanova T.M., Koshel O.Y. (2022). Effects Of Different Natural Food Coloring Additions On The Quality Of Chicken Sausage. J. Chem. Technol..

[143] Öztürk-Kerimoğlu B., Serdaroğlu M. (2019). Powder/Gelled Inulin and Sodium Carbonate as Novel Phosphate Replacers in Restructured Chicken Steaks. J. Food Process Preserv..

[144] Ayuso P., Peñalver R., Quizhpe J., Rosell M.d.L.Á., Nieto G. (2024). Broccoli, Artichoke, Carob and Apple By-Products as a Source of Soluble Fiber: How It Can Be Affected by Enzymatic Treatment with Pectinex® Ultra SP-L, Viscozyme® L and Celluclast® 1.5 L. Foods.

[145] Tahiri M., Johnsrud C., Steffensen I.L. (2023). Evidence and Hypotheses on Adverse Effects of the Food Additives Carrageenan (E 407)/Processed Eucheuma Seaweed (E 407a) and Carboxymethylcellulose (E 466) on the Intestines: A Scoping Review. Crit. Rev. Toxicol..

[146] Tomasevic I., Djekic I., Font-i-Furnols M., Terjung N., Lorenzo J.M. (2021). Recent Advances in Meat Color Research. Curr. Opin. Food Sci..

[147] Ramesh M., Muthuraman A. (2018). Flavoring and Coloring Agents: Health Risks and Potential Problems. Natural and Artificial Flavoring Agents and Food Dyes.

[148] Baldin J.C., Michelin E.C., Polizer Y.J., Rodrigues I., de Godoy S.H.S., Fregonesi R.P., Pires M.A., Carvalho L.T., Fávaro-Trindade C.S., de Lima C.G. (2016). Microencapsulated Jabuticaba (Myrciaria Cauliflora) Extract Added to Fresh Sausage as Natural Dye with Antioxidant and Antimicrobial Activity. Meat Sci..

[149] Orădan A.C., Tocai A.C., Rosan C.A., Vicas S.I. (2024). Fruit Extracts Incorporated into Meat Products as Natural Antioxidants, Preservatives, and Colorants. Processes.

[150] He Y., Wang B., Wen L., Wang F., Yu H., Chen D., Su X., Zhang C. (2022). Effects of Dietary Fiber on Human Health. Food Sci. Hum. Wellness.

[151] Pathania S., Kaur N. (2022). Utilization of Fruits and Vegetable By-Products for Isolation of Dietary Fibres and Its Potential Application as Functional Ingredients. Bioact. Carbohydr. Diet. Fibre.

[152] Reynolds A.N., Akerman A., Kumar S., Diep Pham H.T., Coffey S., Mann J. (2022). Dietary Fibre in Hypertension and Cardiovascular Disease Management: Systematic Review and Meta-Analyses. BMC Med..

[153] Costabile G., Griffo E., Cipriano P., Vetrani C., Vitale M., Mamone G., Rivellese A.A., Riccardi G., Giacco R. (2018). Subjective Satiety and Plasma PYY Concentration after Wholemeal Pasta. Appetite.

[154] Du Y., He C., An Y., Huang Y., Zhang H., Fu W., Wang M., Shan Z., Xie J., Yang Y. (2024). The Role of Short Chain Fatty Acids in Inflammation and Body Health. Int. J. Mol. Sci..

[155] Alvandi E., Wong W.K.M., Joglekar M.V., Spring K.J., Hardikar A.A. (2022). Short-Chain Fatty Acid Concentrations in the Incidence and Risk-Stratification of Colorectal Cancer: A Systematic Review and Meta-Analysis. BMC Med..

[156] Mehta N., Ahlawat S., Sharma D., Yadav S., Krishnakanth M. (2018). Development of Dietary Fiber Rich Chicken Meat Rolls and Patties Using Rice Bran. Fleischwirtsch. Int. J. Meat Prod. Meat Process..

[157] Jovanovichs M.R.C., Pinton M.B., Correa L.P., Pedro D., Mallmann C.A., Wagner R., Cichoski A.J., Lorenzo J.M., Teixeira A.J.C., Campagnol P.C.B. (2023). Replacing Animal Fat with Gels of Psyllium Fiber and Combined Linseed Oil–Psyllium Fiber in Salamis: Impacts on Technological, Nutritional, Oxidative, and Sensory Properties. Foods.

[158] Salejda A.M., Olender K., Zielińska-Dawidziak M., Mazur M., Szperlik J., Miedzianka J., Zawiślak I., Kolniak-Ostek J., Szmaja A. (2022). Frankfurter-Type Sausage Enriched with Buckwheat By-Product as a Source of Bioactive Compounds. Foods.

[159] Savadkoohi S., Hoogenkamp H., Shamsi K., Farahnaky A. (2014). Color, Sensory and Textural Attributes of Beef Frankfurter, Beef Ham and Meat-Free Sausage Containing Tomato Pomace. Meat Sci..

[160] D’Ambra K., Minelli G., Lo Fiego D. (2023). Pietro Effect of Hazelnut Skin and Dry Tomato Peel on the Oxidative Stability, Chemical and Sensory Properties of Pork Burgers during Refrigerated Storage. Food Packag. Shelf Life.

[161] Tarasevičienė Ž., Čechovičienė I., Paulauskienė A., Gumbytė M., Blinstrubienė A., Burbulis N. (2022). The Effect of Berry Pomace on Quality Changes of Beef Patties during Refrigerated Storage. Foods.

[162] Babaoğlu A.S., Unal K., Dilek N.M., Poçan H.B., Karakaya M. (2022). Antioxidant and Antimicrobial Effects of Blackberry, Black Chokeberry, Blueberry, and Red Currant Pomace Extracts on Beef Patties Subject to Refrigerated Storage. Meat Sci..

[163] Ayuso P., Quizhpe J., Yepes F., Miranzo D., Avellaneda A., Nieto G., Ros G. (2024). Improving the Nutritional Quality of Protein and Microbiota Effects in Additive- and Allergen-Free Cooked Meat Products. Foods.

[164] Fernández J., Ledesma E., Monte J., Millán E., Costa P., de la Fuente V.G., García M.T.F., Martínez-Camblor P., Villar C.J., Lombó F. (2019). Traditional Processed Meat Products Re-Designed Towards Inulin-Rich Functional Foods Reduce Polyps in Two Colorectal Cancer Animal Models. Sci. Rep..

[165] Singh T., Chatli M.K., Mehta N., Kumar P., Malav O.P. (2015). Effect of Carrot Powder on the Quality Attributes of Fibre-Enriched Spent Hen Meat Cutlets. J. Anim. Res..

[166] Zhang M., Wang Z., Wu J., Lu J., Liu D., Huang Y., Lv G. (2023). Effects of Adding Citrus Fiber with Different Chemical Compositions and Physicochemical Properties on the Cooking Yield of Spiced Beef. LWT.

[167] Powell M.J., Sebranek J.G., Prusa K.J., Tarté R., Powell M.J., Sebranek J.G., Prusa K.J., Tarté R. (2021). Effect of Citrus Fiber Addition on Quality Attributes of Fully Cooked Deli-Style Turkey Breast. Meat Muscle Biol..

[168] Pérez-Burillo S., Mehta T., Pastoriza S., Kramer D.L., Paliy O., Rufián-Henares J.Á. (2019). Potential Probiotic Salami with Dietary Fiber Modulates Antioxidant Capacity, Short Chain Fatty Acid Production and Gut Microbiota Community Structure. LWT.

[169] Zaki E.F. (2018). Impact of Adding Chia Seeds (Salvia Hispanica) on the Quality Properties of Camel Burger “Camburger” during Cold Storage. Int. J. Curr. Microbiol. Appl. Sci..

[170] Kim C.J., Kim H.W., Hwang K.E., Song D.H., Ham Y.K., Choi J.H., Kim Y.B., Choi Y.S. (2016). Effects of Dietary Fiber Extracted from Pumpkin (Cucurbita Maxima Duch.) on the Physico-Chemical and Sensory Characteristics of Reduced-Fat Frankfurters. Korean J. Food Sci. Anim. Resour..

[171] Lobiuc A., Pavăl N.E., Mangalagiu I.I., Gheorghiță R., Teliban G.C., Amăriucăi-Mantu D., Stoleru V. (2023). Future Antimicrobials: Natural and Functionalized Phenolics. Molecules.

[172] Larsson S.C., Ericson U., Dekkers K.F., Arage G., Rašo L.M., Sayols-Baixeras S., Hammar U., Baldanzi G., Nguyen D., Nielsen H.B. (2025). Meat Intake in Relation to Composition and Function of Gut Microbiota. Clin. Nutr..

[173] Thøgersen R., Castro-Mejía J.L., Sundekilde U.K., Hansen L.H., Hansen A.K., Nielsen D.S., Bertram H.C. (2018). Ingestion of an Inulin-Enriched Pork Sausage Product Positively Modulates the Gut Microbiome and Metabolome of Healthy Rats. Mol. Nutr. Food Res..

[174] Thøgersen R., Gray N., Kuhnle G., Van Hecke T., De Smet S., Young J.F., Sundekilde U.K., Hansen A.K., Bertram H.C. (2020). Inulin-Fortification of a Processed Meat Product Attenuates Formation of Nitroso Compounds in the Gut of Healthy Rats. Food Chem..

[175] Pérez-Burillo S., Pastoriza S., Gironés A., Avellaneda A., Pilar Francino M., Rufián-Henares J.A. (2020). Potential Probiotic Salami with Dietary Fiber Modulates Metabolism and Gut Microbiota in a Human Intervention Study. J. Funct. Foods.

[176] Nieto G. (2013). Incorporation of by-products of rosemary and thyme in the diet of ewes: Effect on the fatty acid profile of lamb. Eur. Food Res. Technol..

[177] Nieto G., Banón S., Garrido M. (2012). Administration of distillate thyme leaves into the diet of Segureña ewes: Effect on lamb meat quality. Animal.

[178] Banón S., Díaz P., Nieto G., Castillo M., Álvarez D. (2008). Modelling the Yield and Texture of Comminuted Pork Products Using Color and Temperature. Effect of Fat/Lean Ratio and Starch. Meat Sci..

